# Structural basis for hepatitis E virus neutralization by potent human antibodies

**DOI:** 10.1126/sciadv.adu8811

**Published:** 2025-05-07

**Authors:** Luis M. Molinos-Albert, Eduard Baquero, Cyril Planchais, Virginie Doceul, Hicham El Costa, Estelle Mottez, Vincent Mallet, Stanislas Pol, Matthew L. Albert, Nicole Pavio, Cécile Alanio, Jordan D. Dimitrov, Hugo Mouquet

**Affiliations:** ^1^ Humoral Immunology Unit, Institut Pasteur, Université Paris Cité, 75015 Paris, France.; ^2^ NanoImaging Core Facility, Centre de Ressources et Recherches Technologiques (C2RT), Institut Pasteur, Université Paris Cité, 75015 Paris, France.; ^3^ UMR Virology, École Nationale Vétérinaire d'Alfort, INRAE, ANSES, 94704 Maisons-Alfort, France.; ^4^ Toulouse Institute for Infectious and Inflammatory Diseases (Infinity), INSERM-CNRS-University Toulouse III, 31024 Toulouse, France.; ^5^ Human Immunology Center, Immunobiology of Dendritic Cells Unit, Institut Pasteur, 75015 Paris, France.; ^6^ Groupe Hospitalier Cochin Port Royal, DMU Cancérologie et Spécialités Médico-Chirurgicales, Service d'Hépatologie, AP-HP Centre, Université Paris Cité, 75014 Paris, France.; ^7^ Centre de Recherche des Cordeliers, INSERM, Sorbonne Université, Université de Paris, 75006 Paris, France.

## Abstract

Antibodies targeting the hepatitis E virus (HEV) surface capsid protein (CA) are essential for infection control and resolution, yet their molecular and functional attributes remain largely elusive. We characterized 144 human HEV-CA–specific monoclonal antibodies cloned from the memory B cells of HEV-exposed individuals. Most human anti-CA antibodies cross-reacted with all HEV genotype variants, and a subset also recognized the zoonotic rat hepatitis E virus. HEV antibody repertoire was diverse and contained highly potent neutralizing antibodies binding to the CA protruding (P) domain. Structural analyses of CA protein complexed with three potent and broad HEV antibodies uncovered a neutralizing site located on monomeric P domain loops at the apex of the viral spike. These findings provide valuable insights into the protective humoral response to HEV and offer a framework for the rational design of HEV vaccines and immunotherapies.

## Teaser

Potent and broad human HEV antibodies reveal a previously unknown neutralizing site for vaccine design.

## INTRODUCTION

Hepatitis E virus (HEV) infection is the leading cause of acute hepatitis, annually affecting ~20 million people worldwide and resulting in 30,000 to 40,000 deaths (*1*). HEV infection is often self-limiting, but it can lead to fatal acute liver failure in pregnant women and chronic hepatitis in immunocompromised individuals (*2*, *3*). HEV belongs to the *Paslahepevirus* genus of the *Hepeviradae* family and comprises eight genotypes to date (*4*). HEV-1 to HEV-4 are the primary genotypes responsible for human infections. HEV-1 and HEV-2 are human restricted and transmitted by the fecal-oral route via contaminated water causing epidemics in areas with poor sanitation. In contrast, HEV-3 and HEV-4 are foodborne viruses responsible for outbreaks worldwide (*3*, *5*).

HEV is a small nonenveloped virus with a single-stranded positive-sense RNA genome containing three major open reading frames (ORF1 to ORF3). HEV is shed into feces as a “naked” viral form, also found in the bile, but circulates in the blood as quasi-enveloped virions coated by host-derived membranes (*6*). The viral capsid protein (CA) is encoded by the ORF2 and assembles in a *T* = 3 icosahedral symmetry with 90 homodimeric subunits to form the shell of the viral particle (*7*). The HEV-CA monomer is composed of the protruding (P), the shell (S), and middle (M) domains. The S and M domains associate tightly around the particle’s threefold axis, whereas the P domain, linked to the M domain, extends from the virion surface as a dimeric spike (*7*, *8*). The P domain is essential for virus-host interactions, mediating preattachment to heparan sulfate proteoglycans and binding to unidentified receptor(s) on the cell surface (*9*, *10*).

HEV antibodies elicited in response to infection play a crucial role in clearing the virus and protecting from liver disease (*11*, *12*). Long-lasting humoral immune memory induced by infection, including HEV-specific circulating antibodies and memory B cells, certainly plays a key protective role against reinfections (*13*, *14*). Antibodies neutralizing HEV target the viral CA protein, specifically the P domain, and inhibit de novo infection by either blocking the attachment of naked HEV virions to target cells or inducing structural disruption of the virus (*14*, *15*). Serological studies shed some light on the HEV antibody response, such as the identification of conformational neutralizing epitopes within an immunodominant region of the P domain (*11*, *12*). HEV neutralizing monoclonal antibodies produced from immunized mice were also described (*16*, *17*). Notably, 8G12, a murine neutralizing antibody targeting an epitope at the dimeric P interface was found to cross-react with the four human HEV genotypes (*17*).

Despite existing serological data on the HEV antibody response, the molecular details, and structure-function relationships of human HEV antibodies remain elusive. We thus set out to unravel the memory antibody response to the viral capsid antigen and structural basis of broad and potent neutralization of HEV. Here, we present a detailed characterization of 144 human HEV-CA antibodies cloned from memory B cells of HEV seroconverters.

## RESULTS

### Human HEV memory B cell antibodies

**Fig. 1.**
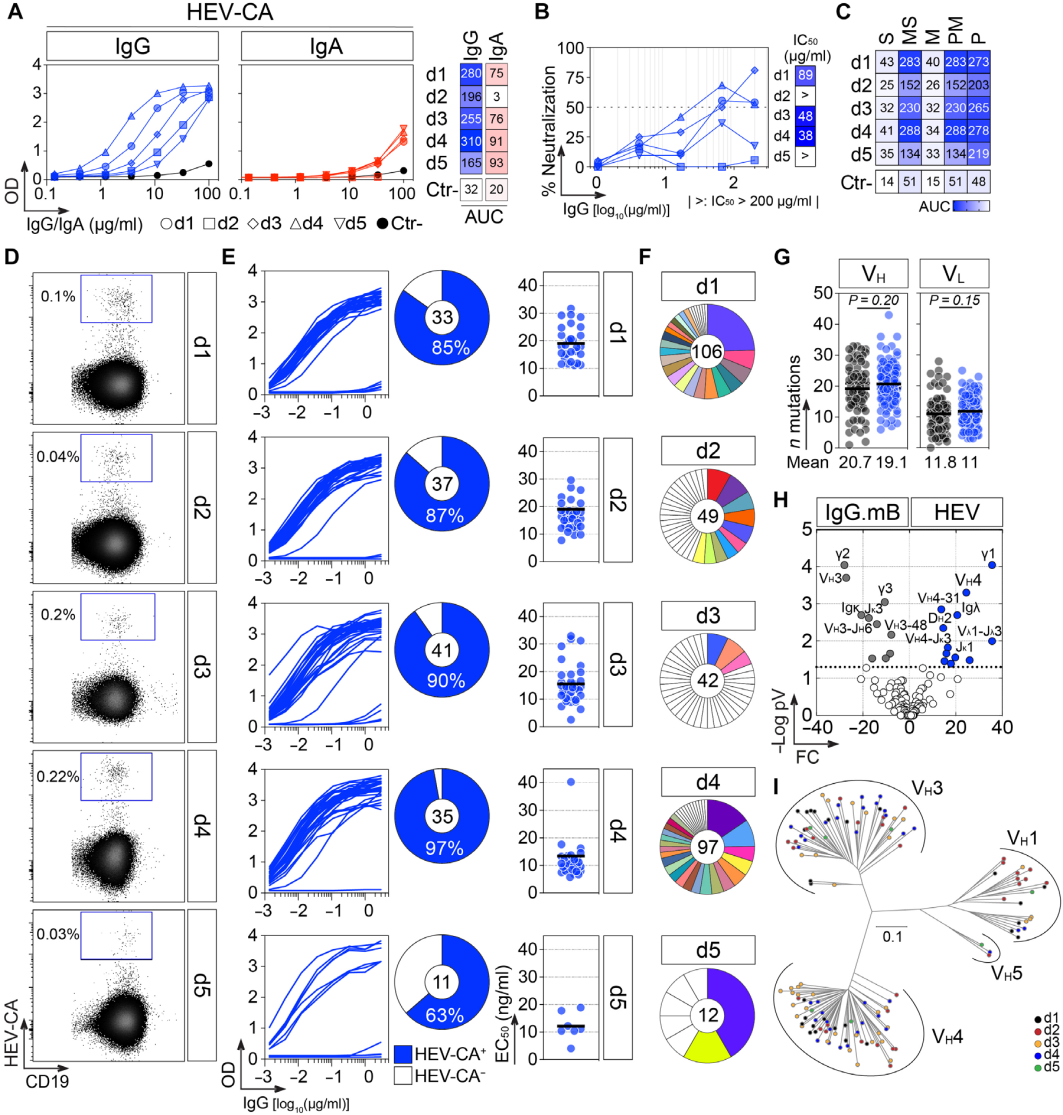
Human HEV memory B cell antibodies. (**A**) ELISA graphs showing the reactivity of purified serum IgGs (blue) and IgAs (red) from HEV-exposed donors against HEV-CA protein (left). The heatmap comparing the area under the curve (AUC) values derived from the ELISA titration curves (right). The black line corresponds to the negative control (Ctr-, seronegative donor). (**B**) Graph comparing the in vitro neutralization activity of purified IgGs against naked HEV virions (HEV-3c) (left). Calculated IC_50_ values are presented on the heatmap (right). (**C**) Heatmap comparing the AUC values for the ELISA binding of purified IgGs to HEV-CA domain proteins. (**D**) Flow cytometric plots showing the percentage of circulating blood HEV-CA^+^ IgG memory B cells in HEV-exposed individuals. (**E**) ELISA graphs showing the reactivity of the HEV memory B cell antibodies against HEV-CA protein (left). The pie charts (middle) show the proportion of antibodies binding to the HEV-CA protein. The number of tested antibodies is indicated in the center of the pie chart. EC_50_ values calculated from the titration curves are represented in the dot plots (right). (**F**) Pie charts showing the distribution of clonally expanded (colored) and unique (white) IgG^+^ B cell clones (306 total sequences) with confirmed HEV-CA specificity, with only one representative IgG antibody tested per clonal family, as presented in (E) (*n* = 138). The total number of clones, including clonal variants, is indicated in the center of the pie chart. (**G**) Dot plots comparing the number of V_H_ and V_L_ gene mutations in HEV-CA (*n* = 138, blue) and control (*n* = 71, black) IgG antibodies. (**H**) Volcano plot analysis comparing the Ig gene repertoire of HEV-CA–specific IgG^+^ B cells and total IgG^+^ memory B cells from healthy individuals (IgG.mB) (*20*). FC, fold change. (**I**) Dendrogram showing the maximum likelihood estimation of the similarity level between V_H_ amino acid sequences of HEV antibodies from all donors.

To characterize the memory B cell antibody response to HEV in individuals who seroconverted after infection, we first produced a soluble recombinant HEV-CA protein [genotype 3 (*7*), HEV-ORF2_126-601_] that forms homodimers having the potential to assemble into capsid viral particles (HEV-LP/*T* = 1) (fig. S1, A and B) (*18*). The five selected HEV-exposed donors showed serum immunoglobulin G (IgG) antibody binding to HEV-CA protein by enzyme-linked immunosorbent assay (ELISA) [median effective concentration (EC_50_) range (5.3 to 53.7 μg/ml)] but low IgA seroreactivity (Fig. 1A and table S1). Purified serum IgG antibodies from donors d1, d3, and d4 neutralized in vitro naked genotype 3 HEV (3c strain) with median inhibitory concentration (IC_50_) values ranging from 38 to 89 μg/ml, whereas no neutralization was evidenced for individuals with weaker HEV-CA IgG titers (d2 and d5) (Fig. 1B). Binding analyses of the purified serum IgGs using recombinant protein domains [protruding (P), middle (M), and shell (S), P plus M (PM), and M plus S (MS)] showed strong antibody responses against P, PM, and MS but no or low IgG reactivity against the S or M proteins alone (Fig. 1C and fig. S1C). Competition experiments for P or MS antigen recognition by serum IgG antibodies in the presence of PM protein showed a decreased reactivity against P only, suggesting that most anti-MS IgGs target epitopes overlapping the M and S domains or the S domain alone (fig. S1D). No distinct HEV-CA immunoreactive peptides were unambiguously identified; however, two peptides located at the N and C termini of the M domain exhibited slightly higher antibody reactivity for some donors (fig. S1E). This suggests that serum HEV-CA antibodies predominantly recognize discontinuous epitopes, consistent with a previous report (*14*).

Peripheral blood B cells isolated from the five donors were stained with fluorescently labeled HEV-CA dimers and analyzed by flow cytometry. HEV-CA–reactive IgG^+^ and IgA^+^ B cells had frequencies ranging from 0.026 to 0.23% and 0.035 to 0.060%, respectively (Fig. 1D and fig. S2A) and were mainly resting memory B lymphocytes (CD19^+^CD27^+^CD21^+^; figs. S1, F to H, and S2B). From 1065 HEV-CA–binding memory B cells captured by single-cell flow cytometric sorting, 684 heavy (IgH) and 693 light (IgL) variable domain genes were amplified by reverse transcription polymerase chain reaction (RT-PCR). From the 443 matched antibody sequences, we produced a total of 172 unique monoclonal antibodies (157 IgG and 15 IgA) by recombinant expression cloning (*19*). A total of 138 IgG [87% (63 to 97%)] and 6 IgA [40% (0 to 6); all cloned from donor d1] antibodies strongly bound to HEV-CA protein by ELISA [mean EC_50_ = 16 (2 to 96) ng/ml; Fig. 1E, fig. S2C, fig. S3 and table S2]. Fifty-seven of the 138 anti-HEV-CA antibodies (41%) were part of clonally expanded B cell lineages, which were mainly found in donors d1 and d4 (75 and 65%, respectively) (Fig. 1F). Only one clonotype was identified among HEV IgA antibodies (fig. S2D).

### Antibody repertoire and reactivity profile of HEV antibodies

Immunoglobulin genes of anti-HEV antibodies were somatically mutated at a similar level to those from IgG^+^ memory B cells of healthy donors (IgG.mB) (*20*) (20.7 versus 19.1 nucleotide mutations, *P* = 0.20 for IgH and 11.8 versus 11, *P* = 0.15 for IgL) (Fig. 1G, fig. S4A, and table S2). Comparison of HEV IgG gene features with IgG.mB revealed a substantial enrichment of V_H_4 (*P* = 0.0015) and J_K_1 (*P* = 0.0084) gene usage (Fig. 1H and fig. S4, B to D). Notably, HEV antibodies showed a strong increase in V_H_4-31 gene usage (15.2% versus 1.4% for IgG.mB, *P* = 0.0015; Fig. 1H and fig. S4C). A decreased use of V_H_3-J_H_6 gene rearrangements and a higher expression frequency of IgG1 subclass were also found in HEV antibodies (Fig. 1H and fig. S4, E and F). Notably, HEV antibodies had a strong bias toward kappa light chain (Igκ) usage (83% versus 62% for IgG.mB, *P* = 0.002), particularly those from donors d2 and d4 that all expressed an Igκ (Fig. 1H and fig. S4F). Maximum likelihood estimation of V_H_ amino acid similarity revealed the diversity of the HEV antibody gene repertoire, with sequences shuffled among donors and clustering based on V_H_ family usage (Fig. 1I). Unlike many other antiviral antibodies (*21*, *22*), there was no apparent interindividual sequence convergence indicative of a stereotyped or public antibody response.

**Fig. 2.**
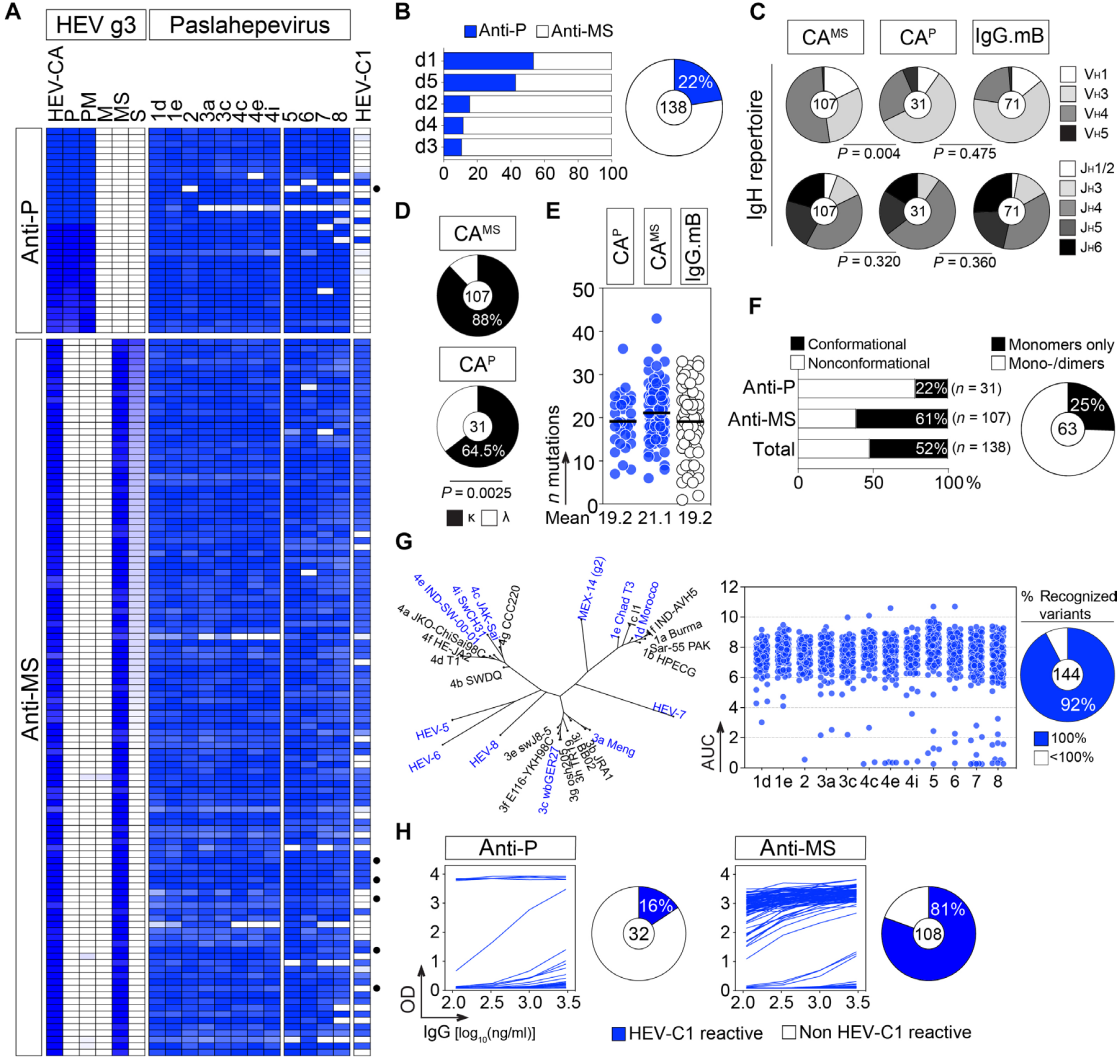
Reactivity profiles of human HEV antibodies and their corresponding immunoglobulin gene repertoire. (**A**) Heatmap showing the reactivity of anti-HEV-CA IgG and IgA memory B cell antibodies (*n* = 144) against HEV g3-2712 capsid domains, *Paslahepevirus* HEV-CA (genotypes 1d to 8) and *Rocahepevirus* CA (HEV-C1) proteins, with color-coded cells according to the values shown in table S2. Black dots on the right indicate IgA antibodies. (**B**) Bar graph and pie chart showing the proportion of IgG antibodies (*n* = 138) recognizing the P domain for each HEV-exposed donor (left) and in total (right), respectively. (**C**) Pie charts comparing the distribution of V_H_ and J_H_ gene usage of anti-CA P domain (CA^P^), anti-CA MS domains (CA^MS^) and control (IgG.mB) IgG memory B cell antibodies. (**D**) Pie charts comparing the κ- versus λ-Ig chain usage of CA^P^ and CA^MS^ IgG antibodies. Groups in [(C) and (D)] were compared using 2 × 5 and 2 × 2 Fisher’s exact test, respectively. (**E**) Dot plots comparing plots comparing the number V_H_, Vκ, and Vλ gene mutations of CA^P^, CA^MS^, and control (IgG.mB, white) IgG antibodies. (**F**) Bar graph showing the proportion of IgGs recognizing strictly conformational HEV-CA epitopes on as shown in fig. S5 (left). Pie chart showing the proportion of IgGs binding to HEV-CA monomers only or to both monomeric and dimeric proteins as determining by immunoblotting (fig. S5) (right). (**G**) Dot plot (right) shows the binding of IgG and IgA antibodies (*n* = 144) to CA proteins from different *Paslahepevirus* genotypes in the dendrogram (left; generated from *Paslahepevirus* CA protein alignment). Dots correspond to ELISA AUC values shown in fig. S3. Pie chart (right) shows the percentage of antibodies binding to all genotypes (blue-colored slice). (**H**) ELISA graphs showing the binding of P-reactive (left) and non–P-reactive (right) IgG antibodies against the *Rocahepevirus* HEV-C1 protein. Pie charts showing the proportion of antibodies recognizing HEV-C1 are shown on the side.

Our ELISA mapping analyses identified 31 IgGs (22.5%, *n* = 138) and 1 IgA (17%, *n* = 6) binding to the P domain (Fig. 2, A and B, and figs. S2E and S3). Thus, except for donor d1 (53.6%, *n* = 28), a minority of HEV antibodies targeted the P domain (d2, 15.6%, *n* = 32; d3, 10.8%, *n* = 37; d4, 11.7%, *n* = 34; d5, 42.8%, *n* = 7) (Fig. 2B and table S2). Non–P-reactive antibodies bound strongly to HEV MS protein, whereas only a moderate if any reactivity to M and S domains alone was detected (Fig. 2A and fig. S3). This is consistent with the serum IgG mapping analyses and confirms the immunodominance of epitopes dependent on both domains. MS-reactive antibodies more frequently used V_H_4 genes (i.e., V_H_4-31) and Igκ [88% versus 64% for anti-P], whereas anti-P were enriched in V_H_3-30–expressing antibodies (Fig. 2, C and D, and fig. S4, H and I). HEV antibodies targeting the P domain exhibited slightly higher levels of somatic mutations compared to those binding to other regions of the CA protein, although this difference did not reach statistical significance (19.2 versus 21.1 nucleotide substitutions, *P* = 0.15; Fig. 2E). Immunoblotting analyses showed that half of the HEV IgG antibodies (52%, *n* = 138) completely lost their reactivity against the denatured capsid protein (fig. S5), indicating that they recognize conformational epitopes. Disruption of capsid conformation more frequently affected the reactivity of anti-MS than anti-P antibodies (61% versus 22%, respectively), indicating a higher prevalence of MS-specific antibodies targeting discontinuous epitopes (Fig. 2F and fig. S5). Most conformation-independent antibodies (75%, *n* = 63) recognized both CA protein dimers and monomers (Fig. 2F and fig. S5).

HEV-5 to HEV-8 variants in the *Paslahepevirus* genus infect animal hosts but can present a zoonotic potential (*23*). To determine whether the human HEV antibodies recognize the different HEV genotypes, we evaluated their binding to purified recombinant capsid proteins from HEV-1 to HEV-8 (fig. S6). The vast majority of the HEV antibodies (92%, *n* = 144) cross-reacted with all capsid variants with an average EC_50_ of 13.6 ng/ml (0.1 to 260 ng/ml) (Fig. 2, A and G, and fig. S3). The HEV-related virus *Orthohepevirus* C1 (or HEV-C1) in the *Rocahepevirus* genus, genetically diverging from the human genotypes (fig. S6), circulates in rodents (*24*). However, it has been reported as an emerging zoonotic pathogen capable of infecting humans (*25*). We found that most HEV antibodies also reacted against the purified HEV-C1 capsid protein by ELISA (Fig. 2, A and H). Anti-MS antibodies bound more frequently than anti-P antibodies (81% versus 16% for anti-P, *P* < 0.0001) (Fig. 2H), which is likely due to the higher degree of sequence conservation in the capsid shell protein region (MS) compared to the more variable P domain.

### Potent HEV neutralizing antibodies

**Fig. 3.**
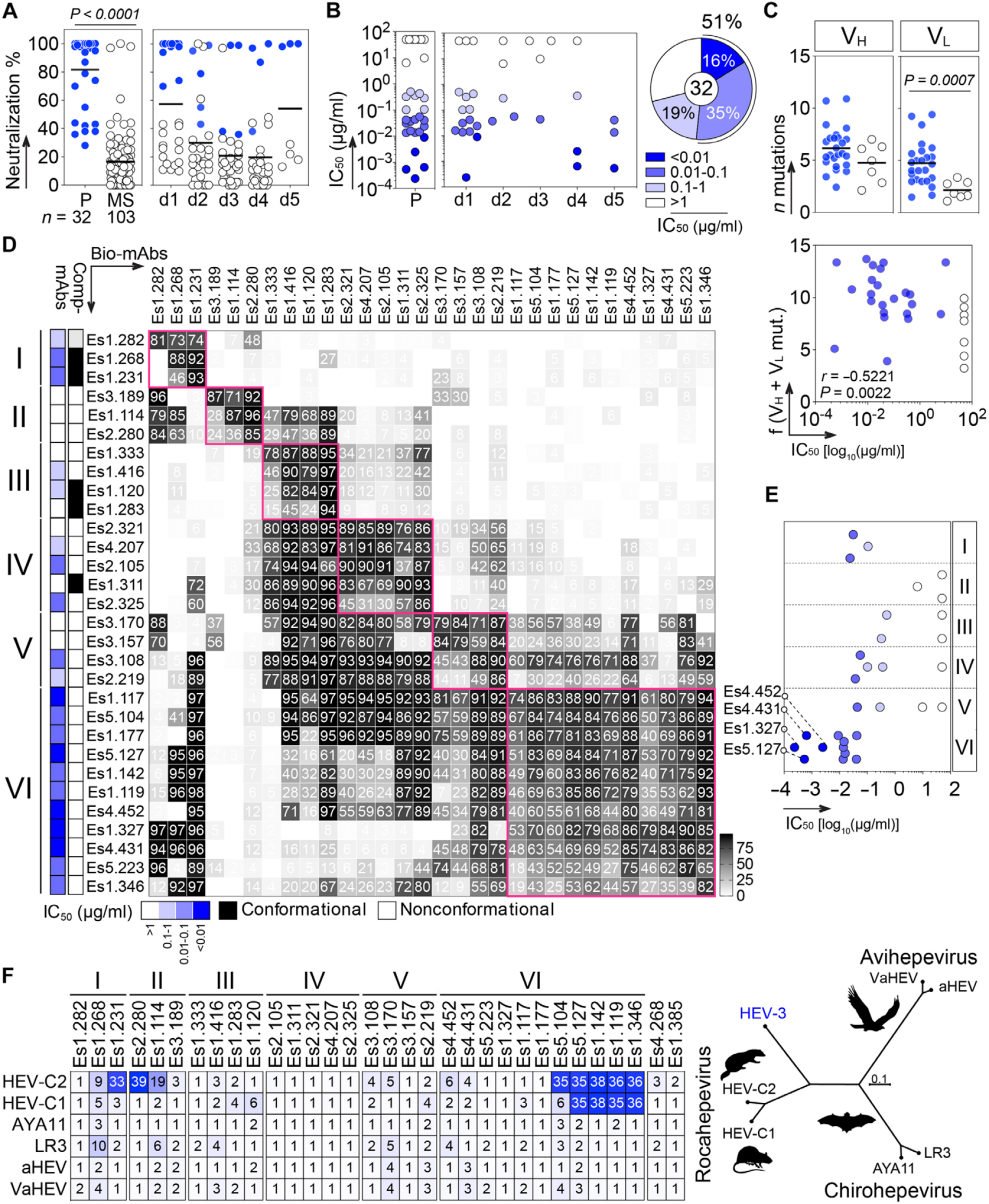
Neutralizing activity of human HEV antibodies. (**A**) Dot plots comparing the percentage of HEV neutralization of human anti-CA antibodies tested in triplicate at 50 μg/ml according to the targeted region (left) and donor (right). Blue-colored dots correspond to P-specific antibodies. (**B**) Dot plots (left) showing IC_50_ values of HEV neutralization for anti-P antibodies segregated according to the donors. Pie chart (right) showing the distribution of anti-P antibodies by IC_50_ values color coded in shades of blue, with darker shades indicating higher potency. (**C**) Dot plots (top) comparing the number of somatic mutations in V_H_ and V_L_ genes between neutralizing and non-neutralizing anti-P antibodies. Antibodies with IC_50_ > 50 μg/ml were considered as non-neutralizing. Groups were compared with the two-tailed Mann-Whitney test. Correlation plot (bottom) showing the frequency of somatic mutations in V_H_ and V_L_ genes [f(V_H_+V_L_ mut.)] versus IC_50_ values of anti-P antibodies. White dots correspond to non-neutralizing antibodies. The two-sided Spearman rho correlation coefficient and corresponding *P* value are shown. (**D**) Competition ELISA heatmap showing the level of binding inhibition to HEV-CA by biotinylated anti-P antibodies (bio-mAbs) in the presence of potential antibody competitor (comp-mAbs). Darker- and white-colored cells indicate high and no or low inhibition, respectively. Antibodies are grouped according to their epitope cluster (I to VI), with colored-coded IC_50_ and the ability to bind strictly conformational versus nonconformational/conformation-sensitive epitopes (left). (**E**) Dot plot showing the distribution of IC_50_ values of anti-P antibodies according with the epitope group. (**F**) Heatmap (left) comparing the reactivity of anti-HEV-CA antibodies to purified recombinant P proteins from selected *Orthohepevirinae* genera (*Rocahepevirus*, *Chirohepevirus*, and *Avihepevirus*), depicted in the dendrogram (left; generated from P domain amino acid alignment). The mean AUCs from duplicate titration values are shown. Cells are color coded according to AUC values, with darker colors indicating high binding and lighter colors representing moderate binding (white: no binding).

We next evaluated the HEV neutralizing activity of the anti-CA IgG antibodies with the in vitro assay using 3c strain naked virions. In line with the role of the capsid P domain in virus-host cell interactions, 78% of anti-P antibodies (*n* = 32) exhibited neutralizing capacity, whereas only few anti-MS antibodies neutralized HEV (3%, *n* = 106) (Fig. 3A). Notably, 22% of anti-P antibodies potently neutralized HEV with IC_50_ values below 10 ng/ml (0.2 to 9 ng/ml), and 35% had an IC_50_ comprised between 13 and 56 ng/ml (Fig. 3B and fig. S7). Together, half of the anti-P antibodies cloned from all five donors efficiently neutralized HEV with IC_50_ < 60 ng/ml (Fig. 3B). Notably, the three anti-P IgGs isolated from donor d5 were potent neutralizers, which contrast with the poor IgG seroneutralization measured in this individual (Figs. 1B and 3B). This suggests a qualitative discordance between the serologic and memory B cell compartments, reminiscent of the seroreversion phenomenon (*11*). Of note, neutralizing anti-P antibodies (IC_50_ < 50 μg/ml) exhibited a higher number of somatic mutations, inversely correlating with antibody IC_50_ values (Fig. 3C). To further map the HEV antibodies, we next performed cross-competition ELISA experiments for their binding to the CA protein. Anti-P antibodies were grouped into six epitope clusters (I to VI) based on the competition profiles obtained (Fig. 3D). Between groups, off-diagonal competition with cluster II, IV, V, and VI antibodies were also observed (Fig. 3D), suggesting physical proximity and/or overlap of the targeted epitopes resulting in steric inhibition or allosteric interferences for antibody binding. All members in the largest group (VI, *n* = 11), which bound to nonconformational epitopes, were highly potent neutralizers [average IC_50_ = 13.9 (0.2 to 42) ng/ml] (Fig. 3, D and E). In contrast, many antibodies in the other groups (I to V) did not neutralize HEV or showed only weak neutralization (Fig. 3, D and E). Most members of group VI blocked the binding of antibodies in groups I, III, and IV nonreciprocally whereas reciprocally inhibiting those in group V. Specifically, antibodies Es1.327, Es4.431, Es5.223, and Es1.346 from group VI blocked the binding of groups V and I members only, suggesting diverse binding fingerprints and/or angles of approach on this capsid region. The four anti-P antibodies cross-reacting with the HEV-C1 capsid (Es1.119, Es1.142, Es1.346, and Es5.127) belonged to the group VI and used the Vλ1-51 gene (table S2). However, group VI antibodies did not display any specific global antibody gene features (table S2). Notably, only some group VI antibodies cross-reacted with both P domain proteins of *Rocahepevirus* (ferret and rat HEV), whereas none of the anti-P antibodies bound to P proteins from the *Chirohepevirus* and *Avihepevirus* genera (Fig. 3F). Group VI top neutralizers (Es1.327, Es4.431, Es4.452, and Es5.127) blocked HEV-CA recognition by the murine anti-P antibodies 8C11 and 8H3 (*26*), and none enhanced 8H3 binding to HEV-CA, which occurs following 8C11’s primary attachment (fig. S8) (*27*). These antibodies were neither polyreactive by ELISA nor bound to self-antigens in human protein microarray and multiplex autoantigen panel, as well as by indirect immunofluorescence on HEp-2 cells (fig. S9).

### Structural characterization of potent HEV neutralizing antibodies

**Fig. 4.**
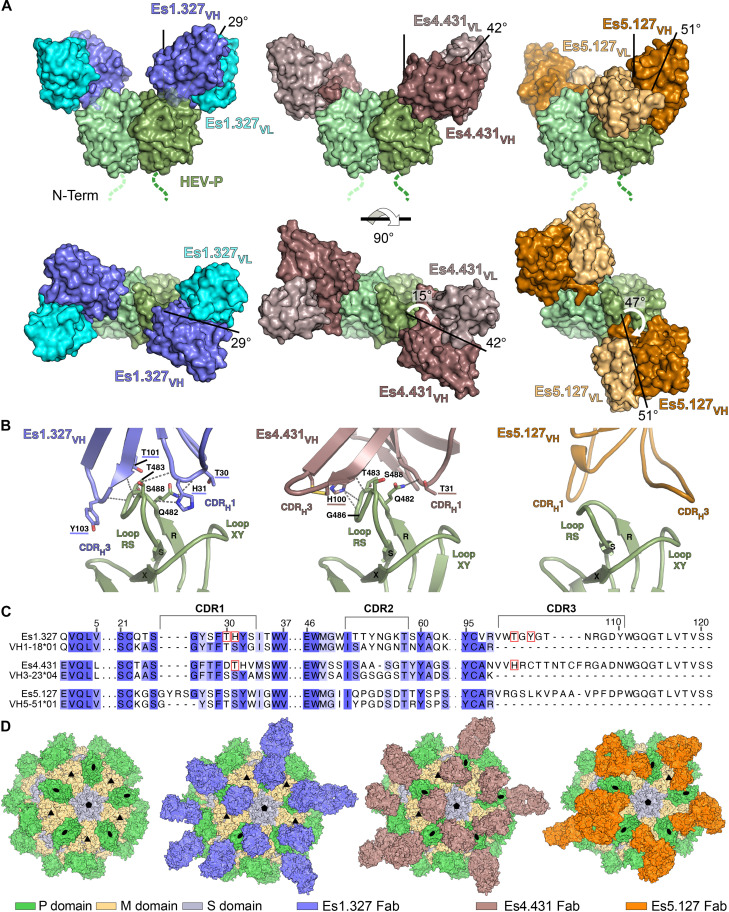
Structure of HEV capsid P domain complexes with Es1.327, Es4.431, and Es5.127. (**A**) Surface representation of cryo-EM models of the HEV-CA P domain in complex with Es1.327, Es4.431, and Es5.127 Fabs. Protomers of the P dimer are colored in shades of green. V_H_ and V_L_ chains of each Fab are highlighted with distinctive colors. The black lines indicate the approach angle. White arrows on top views of the complexes indicate the rotation angle of Es4.431 and Es5.127 Fabs relative to Es1.327 Fab. (**B**) Close-up in ribbon representation of the epitope and paratopes in the P domain–Fab interaction. Hydrogen bonds between chains are represented with dashed lines, and the residues participating in the interactions are indicated. (**C**) Amino acid sequence alignment of Es1.327, Es4.431, and Es5.127 V_H_ and corresponding germline genes. Residues participating in hydrogen bonds indicated in (B) are highlighted with red boxes. (**D**) Binding of Es1.327, Es4.431, and Es5.127 Fabs to HEV viral-like particle (VLP). Surface representation of the crystal structure of *Paslahepevirus* HEV VLP (PDB ID: 3HAG) colored by domains according to (A). Fivefold (pentagon), threefold (triangle), and twofold (oval) symmetry axes are indicated. Fabs in surface representation are shown binding to P dimers around one fivefold and one threefold axes.

To define the neutralizing epitopes on the HEV capsid and their interactions with the most potent human antibodies, we performed cryo–electron microscopy (cryo-EM) of the HEV-CA in complex with antigen-binding fragments (Fab) of the group VI anti-P neutralizers Es1.327, Es4.431, and Es5.127 (Fig. 3E, figs. S7 and S10, and tables S3 to S5). Global refinements yielded well resolved maps of 3.5- and 3.4-Å resolution for Es1.327 and Es4.431 Fab-HEV-CA complexes, respectively (fig. S10). Particles complexed with Es5.127 Fab adopted preferred orientations in the vitrified ice hampering the reconstruction of interpretable maps. A ternary complex with the nonoverlapping Es1.114 Fab helped to obtain low-resolution reconstructions, which despite the density discontinuity, allowed fitting AlphaFold models into density and thus identify the interaction zone of Es5.127 (figs. S10 and S11A). Collectively, the three cryo-EM maps displayed one Fab molecule bound to each protomer of the P dimer (Fig. 4A and fig. S10), whereas S and M domains were not visible due to high flexibility of the connecting linkers.

The structure of the three Fab-HEV-CA complexes revealed that most of the contacts were mediated by the IgH, representing 86 and 80% of the total interface area for Es1.327 and Es4.431, respectively, and recognizing a small, exposed region at the top of the P domain without involving the dimeric interface (Fig. 4A, fig. S11B, and table S6). This region spans only 7.8 and 6.5% of the exposed surface of the P domain in the interaction with Es1.327 and Es4.431, respectively, and involves the loops between β strands R and S (residues 481 to 492) and X and Y (residues 580 to 594) (*8*), the former embedded between the CDR_H_1 and CDR_H_3 loops supporting most of the Fab contacts (Fig. 4B and fig. S11D). The CDR_H_3 loops of Es1.327 (12 residues) and Es4.431 (15 residues) establish hydrogen bonds with the main and side chains of a similar repertoire of P residues including Q482, T483, and S488 (Fig. 4B, fig. S11D, and table S6). Alignment with V_H_ germline genes shows that CDR_H_1 residues involved in RS loop interactions, located at positions H31 and T31 of Es1.327 and Es4.431, respectively, are somatically mutated (Fig. 4C).

Among the three antibodies, Es1.327 exhibited the narrowest approach angle (29°), whereas Es4.431 and Es5.127 displayed wider angles (42° and 51°, respectively) (Fig. 4A). Es5.127 lies nearly perpendicularly to the P domain dimer (Fig. 4A), and the comparison of the position of CDR_H_1 and CDR_H_3 with the other two Fabs showed that they are in opposite directions relative to loops RS and XY, indicating that its Fab flips around 180° to interact with this common interaction zone (Fig. 4, A and B). Modeling of Fab binding to the viral particle showed that Es1.327 and Es4.431 can occupy all available binding sites without clashes between Fabs or affecting the organization of the virion (Fig. 4D). In contrast, the wider approach angle of Fab Es5.127 would cause a clash with the Fabs binding to neighbor P dimers; therefore, Es5.127 can only partially occupy the binding sites available at the surface of the virus, limiting its opsonization potential (Fig. 4D). We conclude that the region recognized by group VI antibodies constitutes a distinct HEV-CA vulnerability site that differs from those previously reported for murine neutralizing monoclonal antibodies or vaccinated individuals (fig. S11C) (*28*, *29*).

### Structural and molecular basis of potent HEV neutralizing antibody cross-reactivity

**Fig. 5.**
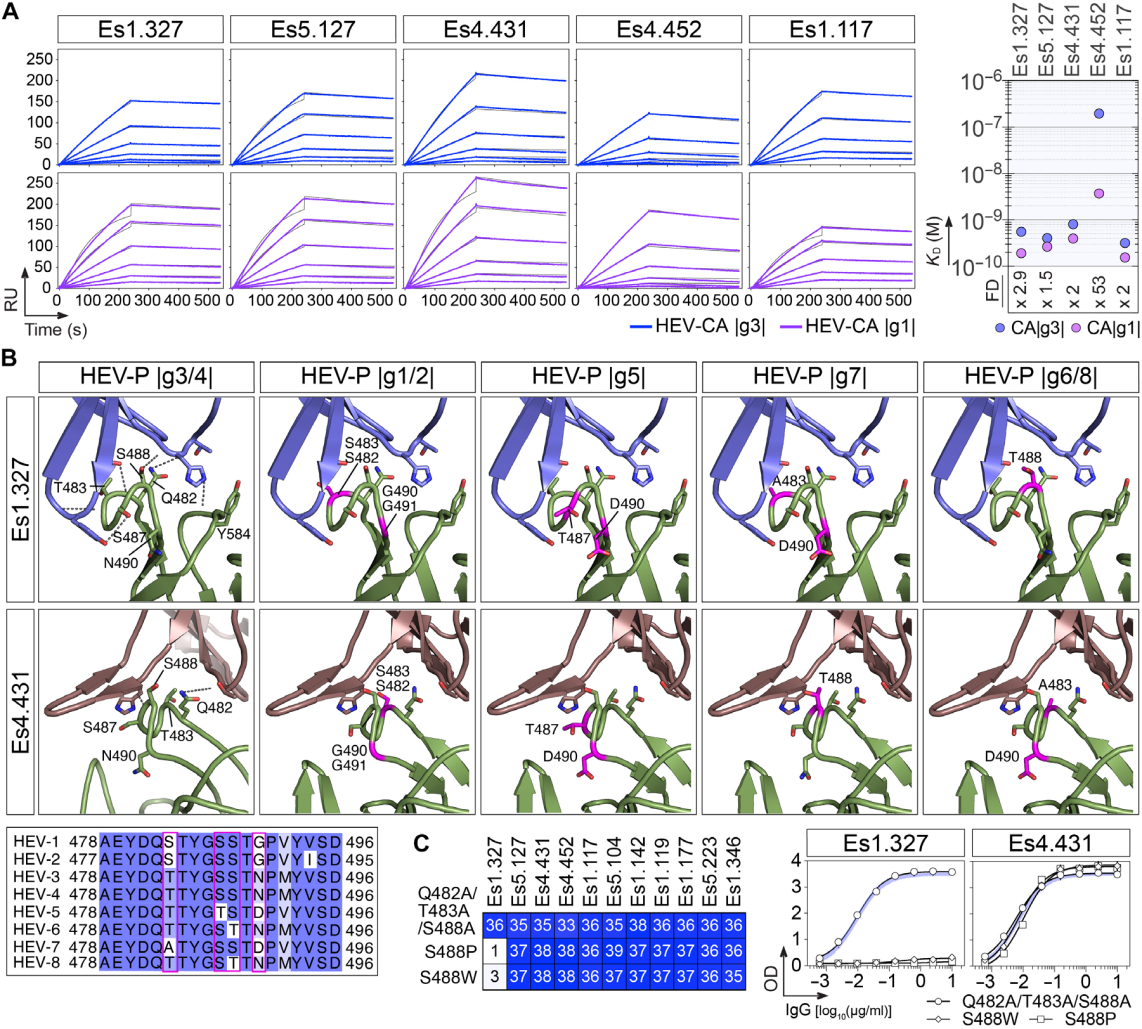
Molecular determinants of cross-genotype binding by group VI anti-P HEV antibodies. (**A**) SPR sensorgrams (left) comparing the apparent affinity of purified neutralizing anti-P IgG antibodies (*n* = 5) for the binding to genotype 1d (purple) and 3a (blue) HEV-CA proteins. Corresponding calculated *K*_D_ values are indicated in the dot plot on the right. RU, response units; FD, fold difference between *K*_D_ values obtained with genotype 1 and 3 HEV-CA proteins. (**B**) Modeling of amino acid variations in the RS loop of the P domain across different HEV genotypes. Ribbon representations of AlphaFold-predicted P domain structures (green) aligned to the cryo-EM model of the HEV P domain genotype 3 in complex with Es1.327 (blue) or Es4.431 Fabs (brown). Dashed lines indicate hydrogen bonds identified in the cryo-EM structures. HEV-CA UniProt accession numbers: P29326, Q03500, Q9YLQ9, A0A348BSN0, BCD83331.1, BFL88677.1, UEC95198.1, and MH410174. Amino acid variants are shown as purple stick representations and highlighted with purple boxes in the sequence alignment (bottom left). (**C**) Heatmap comparing the ELISA binding (as AUC values) of group VI anti-P antibodies to purified recombinant genotype 3 (2712 strain) HEV-CA P domain mutant proteins Q482A/T483A/S488A, S488P, and S488W. The means of assay duplicates are shown. Representative ELISA titration curves of selected antibodies are displayed on the right. Error bars represent the SD of duplicate values. Blue lines indicate the binding to wild-type HEV-CA P domain protein as a comparator.

Despite its genetic diversity, HEV has a single serotype, consistent with conserved neutralizing sites recognized by antibodies from immunized animals and vaccinated individuals (*14*, *15*). In line with this, most human HEV antibodies reported here, including neutralizers, exhibited pan-genotypic reactivity (Fig. 2 and table S2). To further investigate the molecular basis of antibody cross-reactivity, we used surface plasmon resonance (SPR) to measure the affinities of the five most potent group VI anti-P neutralizing antibodies for HEV-CA proteins from genotypes 3 and 1, the latter being among the most divergent among the major genotypes (HEV-1 to HEV-4). Four of the five antibodies (Es1.327, Es5.127, Es4.431, and Es1.117) showed high and comparable affinities for both genotype 3 and 1 HEV-CA proteins (*K*_D_ = 0.16 to 0.81 nM), with a consistent 1.5- to 2.9-fold preference for genotype 1 (Fig. 5A and table S7). In contrast, Es4.452 exhibited a 53-fold higher affinity for genotype 1 compared to genotype 3 (Fig. 5A and table S7). We then examined the cross-reactivity potential of the Es1.327 and Es4.431 antibodies by aligning AlphaFold-generated models of P domains from HEV genotypes 1 to 8 with the cryo-EM structures of the genotype 3 P domain-antibody complexes (Fig. 5B). We found that the RS loop displays conservative amino acid substitutions across genotypes (e.g., Thr-to-Ser and vice versa) and substitutions at positions 490/491, which lie outside the targeted epitopes (Fig. 5B). Therefore, these changes are unlikely to substantially affect antibody binding to the capsid protein in different HEV variants. The binding of group VI anti-P antibodies remained unaffected when key contact residues Q482, T483, and S488 were simultaneously replaced with Ala residues as the interactions primarily involve the main chain of these residues (Fig. 5C). However, mutation of residue S488, located at the tip of the RS loop, which inserts between the CDR_H_1 and CDR_H_3 loops of Es1.327, to either Trp or Pro drastically impaired its binding (Fig. 5C). In contrast, these mutations had no effect on other group VI anti-P antibodies, such as Es4.431, which also binds to the RS loop but engages a different set of interactions due to its longer CDR_H_3 (Fig. 5C). This suggests that only substitutions introducing steric clashes or local conformational changes can disrupt the binding of group VI antibodies to the HEV-CA protein.

**Fig. 6.**
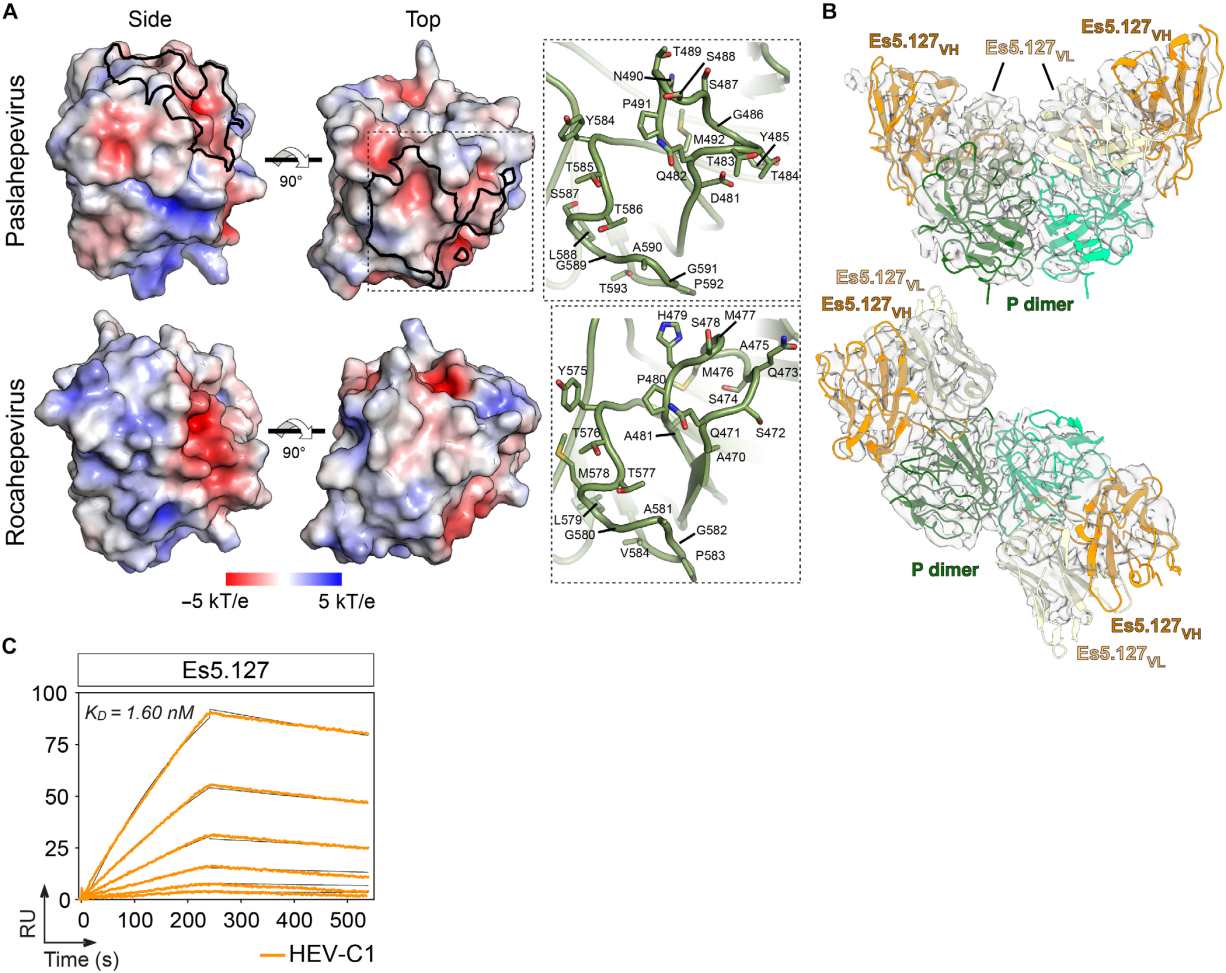
Cryo-EM reconstruction of HEV-C1 P domains in complex with Es5.127. (**A**) Comparison of *Paslahepevirus* (cryo-EM) and *Rocahepevirus* (AlphaFold) HEV P domains. Left and middle panels: Surface representation of P colored by electrostatic potential on a scale from −5 (negative charge) to 5 (positive charge) kT/e. Black line delimits the interaction zone of Es5.127 Fab with *Paslahepevirus* P domain. Right panel: Close-up views of RS and XY loops in ribbon representation of *Paslahepevirus* and *Rocahepevirus* P domain. Side chains of these loops are showed in stick representation. (**B**) Fitting of the model of *Paslahepevirus* P/Es5.127 complex into the cryo-EM reconstruction of *Rocahepevirus* HEV P/Es5.127 complex. (**C**) SPR sensorgram showing the apparent affinity of the anti-HEV P domain IgG antibody Es5.127 for binding to purified HEV-C1 protein. The corresponding calculated *K*_D_ value is indicated in the graph (top left).

The antibody Es5.127 was one of the group VI members cross-reacting with the zoonotic HEV-C1 (table S2). Amino acid sequence alignment and AlphaFold modeling of the HEV-C1 capsid protein revealed a low level of conservation of the RS loop displaying differences in the electrostatic potential of the putative binding site of Es5.127 (Fig. 6A and fig. S6). We obtained a 4.5-Å resolution cryo-EM map of the complex HEV-C1 capsid with Es5.127 Fab (table S8). Our analysis revealed that the HEV-CA/Es5.127 Fab model fits well into this map, providing evidence of a consistent Fab binding despite local differences in the P domain of the two capsid proteins (Fig. 6B). Es5.127 showed high affinity for HEV-C1 by SPR, with a *K*_D_ similar to genotype 3 HEV-CA (0.41 nM versus 1.6 nM) (Fig. 6C and table S7). Future investigations would be needed to determine the neutralizing capacity of Es5.127 against HEV-C1.

## DISCUSSION

The molecular and functional attributes of human antibodies targeting the HEV capsid antigen have remained elusive. Here, we characterized 144 human anti-HEV-CA monoclonal antibodies cloned from the memory B cells of HEV seroconverted donors. We found that, despite the diversity of the HEV antibody gene repertoire and targeted epitopes, anti-capsid memory B cell antibodies harbor certain features, such as the predominance of Igκ and V_H_4-31 gene usage for those targeting conformational MS domain epitopes. Most antibodies recognize the combined MS domains but not as strongly the individual domains, which could be explained by their tight association, and the preferential exposure of shared conformation-dependent epitopes (*8*). In contrast, a fraction of HEV antibodies (22%) bound to the P domain that contains the minimum antigenic region inducing neutralizing antibodies (*16*, *28*, *30*), mainly on nonconformational epitopes. In agreement, the most potent neutralizing antibodies described here targeted the P domain, with group VI antibodies being the top neutralizers. In our diverse anti-P antibody collection, we did not observe preferential traits such as a V_H_1-69 gene bias and dimeric P conformational epitopes as reported in vaccinated humans (*29*) and immunized or infected animals (*10*, *17*, *28*). Anti-capsid IgG antibody and HEV neutralization levels were high for all donors except for d2 and d5. Still, potent group VI neutralizing antibodies were cloned from blood memory B cells of d5. This is reminiscent of seroreversion described in convalescent HEV individuals (*11*, *31*–*33*) and suggests that anamnestic responses in such immune individuals would trigger the production of protecting neutralizing antibodies. Although we demonstrated high affinity, pan-genotypic binding, and epitope conservation for potent anti-P neutralizers, their neutralizing activity against non-genotype 3 HEV has yet to be determined.

Neutralizing epitopes targeted by potent group VI antibodies, revealed by structural analyses, involve two loops (RS) exposed on P domain monomers at the apex of the capsid spike in a region located outside of the dimerization interface (*8*). Despite that these group VI antibodies recognize a common P region, mainly focused on the RS loop, they showed different binding fingerprints and adopted distinct poses. This suggests that this site can accommodate different types of potent antibodies and therefore is a crucial component to consider for HEV vaccine design. Notably, the region centered around the RS loop has never been identified as an HEV neutralizing site in previous human or immunized animal studies (*28*, *29*). The presence of neutralizing epitopes on the P domain outside of the apical dimerized surface (*7*, *10*) suggests there are alternative ways to neutralize HEV by blocking the preattachment and/or receptor-binding steps. Allosteric effects triggering conformational changes in the capsid protein, ultimately causing the physical disruption of virions, have also been reported (*7*, *34*). The extent to which anti-RS loop antibodies elicit such allosteric effects remains unknown. In contrast to monoclonal antibodies cloned from HEV vaccine recipients (*29*), we identified a correlation between somatic mutation frequency and the neutralization potency of anti-P antibodies, consistent with previous findings for severe acute respiratory syndrome coronavirus 2 (SARS-CoV-2) and human immunodeficiency virus–1 (HIV-1) antibodies (*35*, *36*). However, the contribution of somatic mutations to neutralization potency can vary among anti-P antibodies, as demonstrated in group VI. Es1.327 and Es4.431 displayed S31-mutated antigen-contacting residues in their CDR_H_1, a key feature previously reported for hepatitis C virus (HCV) neutralizing antibodies (*37*). In contrast, Es5.127 antibody exhibited minimal somatic mutation loads and primarily formed contacts with the HEV P domain through germline-encoded residues. Thus, potent HEV neutralizers do not always require extensive B cell affinity maturation and could be effective in a near-germline configuration as previously shown for antibodies neutralizing RSV (respiratory syncytial virus), CMV (cytomegalovirus), HCV, and SARS-CoV-2 (*38*–*41*). Binding modeling on HEV virion predicted that Es5.127 would not bind to all available antigenic sites due to clashes between adjacent IgG molecules, whereas Es1.327 and Es4.431 could occupy all available P domains without affecting virion organization. These distinct modes of binding may influence the extent of target opsonization on infected cells and consequently, the potential Fc-dependent antiviral activity of anti-P neutralizing antibodies. In this context, the Fc-dependent effector functions of HEV antibodies remains to be investigated.

The spillover of pathogens from animal hosts to humans is a major contributor to the emergence of infectious diseases as illustrated for instance with HIV-1, Ebola virus, or SARS-CoV-2 (*42*, *43*). Given the serious potential threats to global health posed by zoonotic pathogens, it is crucial to note that HEV has been ranked among the top 10 infectious agents with a high risk of spillover (*44*). Acquired knowledge on humoral immunity in HEV-exposed humans is therefore crucial to help developing future interventional strategies and facilitating outbreak preparedness (*45*, *46*). In line with the proposed concept of a “common serotype” for the four main genotypes (*11*), we found that HEV antibodies were broadly reactive against all *Paslahepevirus* HEV genotypes, also including the minor zoonotic variants HEV-5 to HEV-8 (*24*). HEV-5 and HEV-8 can experimentally infect cynomolgus macaques, and a case of human infection with HEV-7 has been reported, confirming the potential risk of zoonotic HEV variants (*47*–*50*). The zoonotic threat of rat hepatitis virus HEV-C1 was still debated until the recently described human cases in Hong Kong, central Africa, and Europe (*25*, *51*–*54*). Although HEV and HEV-C1 capsid proteins are antigenically distinct, sharing 64% amino acid identity (*55*, *56*), 66% of the HEV antibodies characterized here strongly cross-reacted with HEV-C1 capsid antigen. The vast majority bound to the more conserved viral shell (MS domains), but a fraction of them recognized the P domain. Notably, all four HEV-C1 anti-P-reactive antibodies, isolated from different donors, belonged to group VI and expressed Vλ1-51 gene. Comparison of cryo-EM maps of HEV and HEV-C1 capsid proteins in complex with Es5.127 revealed equivalent binding approaches. Hence, HEV and HEV-C1 capsid proteins contain common epitopes targeted by potent human neutralizing antibodies. Whether Es5.127 antibody neutralizes HEV-C1 remains to be determined. Together, our data highlight the HEV pan-genotypic cross-protective potential of naturally acquired antibodies to HEV upon recall with another genotype and possibly, against zoonotic transmission. Aside from advancing our understanding of the humoral immune response against hepatotropic viruses, our findings have direct implications for developing effective HEV vaccines and antibody-based immune therapies. HEV vaccination confers protection in recipients, and a vaccine based on p239 capsid protein VLPs holds approved use in China (*15*). Thus, it would be interesting to explore whether vaccine-induced polyclonal serum and memory B cell antibodies include group VI HEV-CA antibodies, particularly Es5.127-type IgGs. Murine neutralizing antibodies (*15*) and the potent human HEV neutralizing antibodies reported in this study recognize the P domain, highlighting its potential as a minimal protective immunogen. However, vaccines based solely on the P protein may lack immunogenicity without a potent adjuvant (*15*). Given the health care benefits provided by antibody therapies in combating infectious diseases (*46*, *57*), and the excellent antiviral properties of Es1.327, Es4.431, and Es5.127, these antibodies may represent promising candidates for prophylactic and/or therapeutic interventions against *Paslahepevirus* and *Rocahepevirus* infections. Long-acting versions of such HEV neutralizing antibodies, with extended half-life, could provide protective immunity for immunocompromised populations and pregnant women.

## METHODS

### Human samples

Already stored plasma and peripheral blood mononuclear cell (PBMC) samples from chronically infected HCV individuals used in this study called ANTIHEV were previously obtained from the Liver Unit of Hôpital Cochin (Paris, France) as part of study protocol C11-33 approved by the INSERM clinical investigation department with ethical approval from the CPP Ile-de-France II, Paris (ClinicalTrials.gov identifier: NCT01534728) (*58*). All selected donors had positive anti-HEV IgG titers as measured using the Wantai HEV IgG ELISA kit [mean value = 11.8 (2.7 to 20.1)]. The main clinical and immunovirological characteristics of these donors are summarized in table S1. All donors gave written consent to participate in this study. Ethical issues have been monitored by the Ethics Board for European contracts, an ad hoc independent Ethics Committee in charge of reviewing periodically sensitive ethical issues in EU-funded research when requested by the EU. All human sera were heat inactivated at 56°C for 60 min. Human IgG and IgA antibodies were purified from donors’ sera by affinity chromatography using Protein G Sepharose 4 Fast Flow (GE Healthcare) and peptide M–coupled agarose beads (InvivoGen), respectively. Purified serum antibodies were dialyzed against phosphate-buffered saline (PBS) using Slide-A-Lyzer Cassettes (30K MWCO, Thermo Fisher Scientific).

### Cell lines

Freestyle 293-F suspension cells (Thermo Fisher Scientific) were maintained in FreeStyle Expression Medium (Thermo Fisher Scientific) and kept under constant shaking at 120 rpm. The human hepatoma-derived cell line HepG2/C3A transfected with a MAVS-specific shRNA (*59*) (MAVS-HepG2 cells) were used for HEV neutralization assay. Cells were maintained in penicillin/streptomycin-containing 10% fetal calf serum–Dulbecco’s modified Eagle’s medium (Thermo Fisher Scientific). All cell lines were cultured at 37°C, with 5% CO_2_ and 75% humidity.

### Antigens and antibody controls

Codon-optimized nucleotide fragments encoding recombinant ORF2 proteins from HEV 3-2712 strain (DQ079627) HEV-CA [ORF2_126-601_], HEV-P [OFR2_455-601_], HEV-PM [HEV-OFR2_320-601_], HEV-M [HEV-OFR2_320-454_], HEV-S [HEV-OFR2_126-319_], HEV-MS [HEV-OFR2_126-454_], 1d Morocco (AY230202), 1e Chad T3 (AY204877), MEX-14 (M74506), 3a Meng (AF082843), 3c wbGER27 (FJ705359), 3-2712 (DQ079627), 4c JAK-Sai (AB074915), 4e IND-SW-00-01 (AY723745), and 4i SwCH31 (DQ450072), HEV-C1 (ADM35750.1), and P domains from HEV-C1 (ADM35750.1), HEV-C2 HEV-4351 (AB890001.1), bat HEV-CA AYA11_F (MW249012.1) and LR3_F (MW249014.1), avian HEV-CA aHEV (NC023425.1), and VaHEV (MG976720.1), as well as HEV 3-2712 Q482A/T483A/S488A, S488P and S488W mutant proteins were synthesized and cloned into pcDNA3.1/Zeo^(+)^ expression vector (Thermo Fisher Scientific). All constructs included Kozak and Igκ leader sequences at the N terminus and AviTag and 10xHis C-terminal tags. Recombinant proteins were produced by transient transfection of exponentially growing Freestyle 293-F suspension cells (Thermo Fisher Scientific) using the polyethylenimine (PEI) precipitation method as previously described (*60*). Proteins and antibodies were purified from culture supernatants by high-performance chromatography using the Ni Sepharose Excel Resin or Protein G Sepharose 4 Fast Flow (GE Healthcare), respectively, according to the manufacturer’s instructions dialyzed against PBS using Slide-A-Lyzer dialysis cassettes (Thermo Fisher Scientific), quantified using NanoDrop One instrument (Thermo Fisher Scientific), and controlled for purity by SDS–polyacrylamide gel electrophoresis (PAGE) using NuPAGE 3 to 12% Bis-Tris gels (Life Technologies) as previously described (*60*). AviTagged purified HEV-LP/*T* = 1 CA protein (genotype 3, 2712 strain) protein was biotinylated using the Enzymatic Protein Biotinylation Kit (Sigma-Aldrich). HEV ORF2 20-nucleotide oligomer overlapping peptides were synthetized by GenScript. Murine anti-P antibodies 8C11 and 8H3 (*26*, *27*) were produced as chimeric human IgG1 antibodies. Control antibodies also include polyreactive and nonpolyreactive ED38 (*61*) and mGO53 (*62*), respectively.

### Single B cell sorting and expression-cloning of antibodies

Peripheral blood human B cells were isolated form donors’ PBMCs by magnetic-activated cell sorting using CD19 MicroBeads (Miltenyi Biotec). CD19^+^ B cells were then stained with LIVE/DEAD fixable dead cell stain kit (Thermo Fisher Scientific) and incubated for 30 min at 4°C with 1 μg of biotinylated HEV-LP/*T* = 1 CA protein. Cells were washed once with 1% fetal bovine serum–PBS [fluorescence-activated cell sorting (FACS) buffer] and incubated for 30 min at 4°C with a cocktail of mouse anti-human antibodies: CD19 A700 (HIB19, BD Biosciences, San Jose, CA), CD21 BV421 (B-ly4, BD Biosciences), CD27 PE-CF594 (M-T271, BD Biosciences), IgG BV786 (G18-145, BD Biosciences), IgA FITC (IS11-8E10, Miltenyi Biotec, Bergisch Gladbach, Germany), and streptavidin R-PE conjugate (Invitrogen, Thermo Fisher Scientific). Stained cells were washed with FACS buffer and resuspended in 1 mM EDTA FACS buffer. Single IgG^+^ and IgA^+^ HEV-CA^+^ B cells were sorted into 96-well using a FACSAria III sorter (Becton Dickinson) as previously described (*63*). Following a lymphocyte and single-cell gating, dead cells were excluded. B cell immunophenotyping analyses were performed using FlowJo software (v10.3, FlowJo LLC, Ashland, OR). Single-cell cDNA synthesis using SuperScript IV reverse transcription (Thermo Fisher Scientific) followed by nested PCR amplifications of IgH, Igκ, and Igλ genes, and sequence analysis for Ig gene features were performed as previously described (*19*). Purified digested PCR products were cloned into human Igγ1-, Igκ-, or Igλ-expressing vectors as previously described (*19*). Recombinant antibodies were produced by transient cotransfection of Freestyle 293-F suspension cells (Thermo Fisher Scientific) using the PEI precipitation method as previously described (*60*). Recombinant human IgG antibodies were purified by protein G affinity chromatography (Protein G Sepharose 4 Fast Flow, GE Healthcare). Purified antibodies were dialyzed against PBS. For competition ELISA experiments, purified antibodies were biotinylated using the EZ-Link Sulfo-NHS-Biotin kit (Thermo Fisher Scientific). Es1.114, Es1.327, Es4.431, and Es5.127 IgH were cloned into a 6xHis-tagged IgG1-Fab expression vector as previously described (*64*). Recombinant Fab fragments were produced by transient cotransfection of Freestyle 293-F suspension cells (Thermo Fisher Scientific), purified using Ni-Sepharose Excel affinity chromatography (Cytiva), and dialyzed against PBS (Gibco).

### ELISAs

ELISAs were performed as previously described (*64*). Briefly, high-binding 96-well ELISA plates (Costar, Corning) were coated overnight with 125 ng per well of purified proteins. After washings, plates were blocked 2 hours with 2% bovine serum albumin, 1 mM EDTA, 0.05% and Tween 20–PBS (Blocking buffer), washed, and incubated with serial dilutions of purified serum IgG or IgA antibodies or monoclonal antibodies in PBS for 2 hours. After washings, plates were revealed by incubation for 1 hour with goat horseradish peroxidase (HRP)–conjugated anti-human IgG or IgA antibodies (Immunology Jackson ImmunoResearch, 0.8 μg/ml final) and by adding 100 μl of HRP chromogenic substrate (ABTS solution, Euromedex), after washing steps. After 1-hour incubation, optical densities were measured at 405 nm (OD_405nm_). For competition ELISAs, HEV-CA (3-2712 strain) coated plates were blocked, washed, incubated for 2 hours with biotinylated antibodies (at a concentration 0.33 nM) in 1:2 serially diluted solutions of antibody competitors in PBS (IgG concentration range from 1 to 133 nM), and developed as described above using HRP-conjugated streptavidin (BD Biosciences) (at 0.8 μg/ml in blocking buffer). Binding to overlapping linear peptides covering the HEV capsid and polyreactivity were performed as previously described (*63*). Experiments were performed using a HydroSpeed microplate washer and Sunrise microplate absorbance reader (Tecan Männedorf, Switzerland). Antibodies were tested in duplicate in at least two independent determinations, which included mGO53 or purified serum IgG and IgA antibodies from healthy donors as negative controls. Polyreactivity ELISA was performed as previously described (*65*). Briefly, high-binding 96-well ELISA plates were coated overnight with 500 ng per well of purified double-stranded DNA, KLH, LPS, Lysozyme, Thyroglobulin, Peptidoglycan from *Bacillus subtilis*, 250 ng per well of insulin (Sigma-Aldrich, Saint-Louis, MO), and 125 ng per well of YU2 HIV-1 Env gp140 protein in PBS. After blocking and washing steps, purified HEV IgG antibodies were tested at 4 μg/ml and three consecutive 1:4 dilutions in PBS. Control antibodies, mGO53 (negative), and ED38 (high positive) were included in each experiment. ELISA binding was developed as described above.

### Multiplex bead-based binding assay

Antibody cross-reactivity to self-antigens was evaluated by multiplex autoantigen-binding assay using the Milliplex Human autoimmune autoantibody panel kit (Millipore) following the manufacturer’s instructions. Briefly, IgG antibodies at a final concentration of 25 μg/ml were incubated with the beads overnight at 4°C. After washings, phycoerythrin (PE)–coupled anti-IgG secondary antibodies were added, and mixtures were incubated at room temperature for 1.5 hours. After a final washing step, readings were made on a Bio-Plex 200 instrument (Bio-Rad). mGO53 and ED38 antibodies were used as negative and positive controls for polyreactivity, respectively.

### HEp-2 cell IFA

Binding of human anti-HEV and control monoclonal IgG antibodies (mGO53 and ED38) to HEp-2 cell-expressing autoantigens were analyzed at 100 μg/ml by indirect immunofluorescence assay (IFA) (ANA HEp-2 AeskuSlides, Aesku.Diagnostics) following the manufacturer’s instructions. IFA sections were examined using the fluorescence microscope Axio Imager 2 (Zeiss, Jena, Germany), and pictures were taken at ×40 magnification with 5000-ms acquisition using ZEN imaging software (Zen 2.0 blue version, Zeiss) at the Photonic BioImaging platform (Institut Pasteur).

### Protein microarrays

All experiments were performed at 4°C using ProtoArray Human Protein Microarrays (Thermo Fisher Scientific). Microarrays were blocked for 1 hour in blocking solution (Thermo Fisher Scientific), washed, and incubated for 1.5 hours with IgG antibodies at 2.5 μg/ml as previously described (*65*). After washings, arrays were incubated for 1.5 hours with AF647-conjugated goat anti-human IgG antibodies (at 1 μg/ml in PBS; Thermo Fisher Scientific) and revealed using a GenePix 4000B microarray scanner (Molecular Devices) and GenePix Pro 6.0 software (Molecular Devices). Fluorescence intensities were quantified using Spotxel software (SICASYS Software GmbH, Germany), and mean fluorescence intensity (MFI) signals for each antibody (from duplicate protein spots) was plotted against the reference antibody mGO53 (nonpolyreactive isotype control) using GraphPad Prism software (v8.1.2, GraphPad Prism Inc.). For each antibody, *Z* scores were calculated using ProtoArray Prospector software (v5.2.3, Thermo Fisher Scientific), and deviation (σ) to the diagonal and polyreactivity index (PI) values were calculated as previously described. Antibodies were defined as polyreactive when PI > 0.21.

### Infrared immunoblotting

Purified recombinant HEV-CA 3-2712 protein was heat denatured at 95°C for 5 min in LDS-containing buffer with reducing agent (Invitrogen). Denatured HEV-CA protein (50 μg total) was separated by SDS-PAGE with a NuPAGE 4 to 12% Bis-Tris gels, electrotransferred onto nitrocellulose membranes, and saturated in PBS-0.05% Tween 20 (PBST)–5% dry milk. Membranes were incubated with HEV-CA antibodies (1 μg/ml) and mouse anti-6xHis antibody (1 μg/ml; BD Biosciences) in PBST–5% dry milk for 2 hours. After washing with PBST, membranes were incubated for 1 hour with 1:25,000 diluted Alexa Fluor 680–conjugated donkey anti-human IgG (Jackson ImmunoResearch) and 1:25,000 diluted IR Dye 800CW–conjugated goat anti-mouse IgG (Li-COR Biosciences) in PBST–5% dry milk. Membranes were washed and examined with the Odyssey Infrared Imaging system (LI-COR Biosciences).

### HEV neutralization assay

HEV-3c viral suspensions were generated using fecal samples from infected pigs as previously described (*66*). Viral stock was prepared at 4 × 10^6^ genomic equivalents (GE)/ml to obtain 2 × 10^5^ GE per well. One day before infection, 20,000 MAVS-HepG2 cells per well were seeded into 96-well plates. Serially diluted antibodies and virus were incubated in duplicate or triplicate for 30 min prior to be inoculated onto the cells. After 18 hours, viral suspensions were removed, and cells were washed five times with prewarmed PBS at 37°C before adding 200 μl of fresh growth media supplemented with 2% dimethyl sulfoxide. Cultures were kept at 37°C, 5% CO_2_, for 10 days. Supernatants were harvested and processed to quantify the viral capsid protein and the viral RNA content by ELISA and reverse transcription quantitative polymerase chain reaction (RT-qPCR), respectively. To determine the amount of HEV-CA in culture supernatants, a sandwich ELISA was performed using the nonoverlapping HEV-CA antibodies Es1.114 and Es3.157. Plates were coated overnight with 250 ng per well of the capture antibody (Es1.114). After washings, plates were blocked 2 hours with ELISA blocking buffer, PBST washed, followed by a 2-hour incubation with 50 μl of supernatants or 1:3 serially diluted recombinant HEV-CA as a standard (3 μg/ml starting concentration). After washings, biotinylated antibody Es3.157 (at 0.5 μg/ml) was added for 1 hour, followed PBST washings and a 30-min incubation with 1:1000 diluted HRP-conjugated streptavidin. After washings, plates were revealed as described above. The concentration of virion-free capsid protein was interpolated from the standard curve by nonlinear regression using GraphPad Prism software (v.8.2, GraphPad Prism Inc.). Neutralization percentage was calculated by applying the following formula: 100 − (HEV-CA IgG/HEV-CA no IgG × 100).

### Real-time RT-qPCR

Total RNA was extracted from 150 μl of culture supernatant using the NucleoSpin RNA virus kit (Macherey-Nagel) following the manufacturer’s instructions. RNA was eluted with 50 μl of preheated (70°C) water and immediately stored at −80°C. HEV RNA quantification was performed as previously described (*67*). Briefly, a one-step TaqMan RT-PCR was performed on 2 μl of total RNA (final volume of 20 μl) using the QuantiTect Probe RT-PCR Kit (Qiagen) according to the manufacturer’s instructions. Reverse (JVHEVR, 5′-AGGGGTTGGTTGGATGAA-3′) and forward (JVHEVF, 5′-GGTGGTTTCTGGGGTGAC-3′) primers were used at final concentrations of 0.25 and 0.06 μM, respectively. The probe (JVHEVP, 5′-FAM-TGATTCTCAGCCCTTCGC-MGB-3′) was used at a final concentration of 0.31 μM. Reverse transcription was performed at 50°C for 20 min followed by a denaturation step at 95°C for 15 min. DNA was amplified by 45 cycles at 95°C for 10 s and 58°C for 45 s and a final cooling step at 40°C for 30 s. Sample processing and analysis were performed using a QuantStudio 6 Flex Real-Time PCR System (Thermo Fisher Scientific). Standard quantification curves were generated using HEV RNA obtained from the pcDNA3.1^+^/VHE3f/standard plasmid (*68*). After linearization, 1 μg of the purified plasmid was used for RNA in vitro transcription using the mMESSAGE mMACHINE T7 Transcription Kit (Thermo Fisher Scientific) and then purified using the NucleoSpin RNA Kit (Macherey-Nagel). RNA was immediately aliquoted at a final concentration of 0.5 ×10^12^ copies/μl and stored at −80°C until use.

### Surface plasmon resonance

SPR-based technology (using the Biacore 2000 system: Biacore Cytiva) was used to assess the kinetics of interactions between anti-P monoclonal antibodies and genotype 3a and 1d HEV-CA proteins, as well as HEV-C1 proteins. Protein G sensor chips (ref. no. 29179316, Biacore Cytiva) were used for these experiments. Purified IgG antibodies at a concentration of 10 μg/ml in HBS-EP buffer [10 mM Hepes (pH 7.2), 150 mM NaCl, 3 mM EDTA, and 0.005% Tween 20) were loaded onto the sensor chip channels for a contact time of 1 min. Once the baseline stabilized, HEV-CA proteins at concentrations ranging from 5 or 10 to 0.312 nM (in twofold dilution steps) in HBS-EP buffer were injected over the sensor surface at a flow rate of 30 μl/min. The association and dissociation phases of the antibody-protein interactions were monitored for 4 and 5 min, respectively. All kinetic measurements were conducted at 22°C. Following the injection of purified capsid proteins, the sensor surface was regenerated by exposure to a 10 mM glycine solution at pH 1.5 for 30 s, effectively dissociating IgG molecules from the immobilized protein G. The binding kinetics were determined by global analysis using the Langmuir binding model implemented in the BIAevaluation Software (v4.1.1, Biacore).

### Cryo-EM data collection, refinement, and modeling

Movies of the complexes of purified HEV-CA 3-2712 protein with Fabs Es5.127/Es1.114, Fab Es1.327, and Fab Es4.431 were collected on a Titan Krios transmission electron microscope (Thermo Fisher Scientific) operating at 300 kV equipped with a Gatan K3 direct electron detector and BioQuantum energy filter. Movies were acquired in tiff format in counting mode using EPU automated image acquisition software at a nominal magnification of ×105,000 (0.86 Å/pixel), defocus list of −1.0 to −3.0 μm, and a total dose of ~50 e^−^/Å^2^. The movies for the complex HEV-C1 protein with Fab Es5.127 were collected on a Titan Krios transmission electron microscope (Thermo Fisher Scientific) also operating at 300 kV equipped with a Falcon 4i direct electron detector and Selectris X energy filter. Movies were acquired in EER format with the EPU automated image acquisition software EPU at a nominal magnification of ×215,000 (0.56 Å/pixel), defocus list of −1.0 to −3.0 μm, and a total dose of ~40 e^−^/Å^2^. The raw movies were processed for patch motion correction and patch CTF estimation using cryoSPARC v4.0.3 (*69*). Initial particles were picked using a blob picker, and selected two-dimensional (2D) classes were used for more extensive template picking. After several iterations of 2D classification, three different classes of ab initio models were reconstructed and refined using nonuniform refinement. In the case of the complexes including Fab Es5.127, there was a marked preferred orientation. Picking with Topaz (*70*) helped to recover a set of particles with more orientations allowing reconstruction of more complete ab initio models. The resolution was further improved with local motion correction. Models of the Fabs generated by AlphaFold, and the crystal structure of the P domain [Protein Data Bank (PDB) ID: 3HAG] were fitted into the maps. The models were exanimated and manually corrected using Coot software (*71*) and refined with real-space refinement in the Phenix suite (*72*).

### Statistical analyses

Comparison of the different immunoglobulin gene features between groups were assayed using two-sided 2 × 2 or 2 × 5 Fisher’s exact tests with SISA online tools (http://www.quantitativeskills.com/sisa). The numbers of V_H_, Vκ, and Vλ mutations were compared across groups of antibodies using two-tailed Mann-Whitney test with GraphPad Prism software (v.8.4, GraphPad Prism Inc.). Volcano plot comparing gene features (*n* = 236 parameters) of HEV-CA B cells and control memory B cells was performed using GraphPad Prism software (v.8.4, GraphPad Prism Inc.). The *y* axis indicates the statistics expressed as −log_10_ (*P* values), and the *x* axis represents the differences between the group means for each parameter. The Barnes-Hut implementation of *t*-distributed stochastic neighbor embedding (t-SNE) was computed using FlowJo software (v.10.3, FlowJo LLC, Ashland, OR) with 2000 iterations and a perplexity parameter of 200. Colors represent the density of surface expression markers or cell populations varying from low (blue) to high (red). Phylogenetic tree was built using CLC Main Workbench (Qiagen) on aligned V_H_ sequences using the neighbor-joining method with a bootstrap analysis on 100 replicates.
