# Supplementary material for: Structural basis for hepatitis E virus neutralization by potent human antibodies
Source: Sci Adv. 2025 May 7;11(19):eadu8811. doi: 10.1126/sciadv.adu8811 (PMC12057666; doi:10.1126/sciadv.adu8811)
Supplement: Supplementary file 1 — Figs. S1 to S11 Tables S1 to S8 [file sciadv.adu8811_sm.pdf]

Science Advances
AAAS

Supplementary Materials for

Structural basis for hepatitis E virus neutralization by potent human antibodies

Luis M. Molinos-Albert *et al.*

Corresponding author: Hugo Mouquet, hugo.mouquet@pasteur.fr;
Eduard Baquero, eduard.baquerosalazar@pasteur.fr

Sci. Adv. **11**, eadu8811 (2025)
DOI: 10.1126/sciadv.adu8811

This PDF file includes:

Figs. S1 to S11
Tables S1 to S8

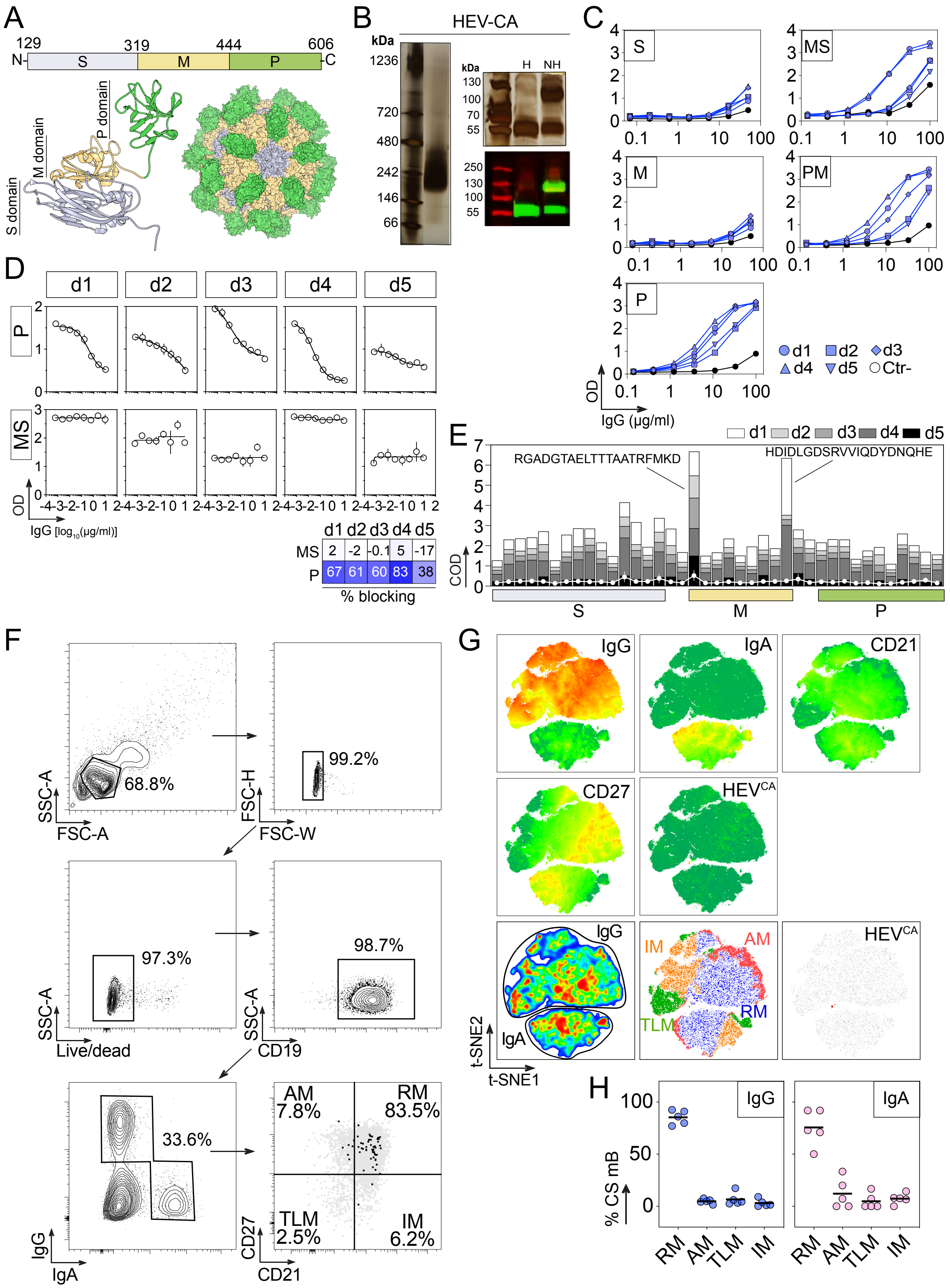
A
129
319
444
606
N-
S
M
P
-C
P domain
M domain
S domain
B
HEV-CA
kDa
1236
720
480
242
146
66
kDa
130
100
70
55
H
NH
250
130
100
55
C
S
MS
M
PM
P
0.1
1
10
100
OD
IgG (µg/ml)
d1
d2
d3
d4
d5
Ctr-
D
d1
d2
d3
d4
d5
P
MS
OD
-4-3-2-10 1 2
IgG [log10(µg/ml)]
d1 d2 d3 d4 d5
MS 2 -2 -0.1 5 -17
P 67 61 60 83 38
% blocking
E
d1
d2
d3
d4
d5
RGADGTAELTTTAATRFMKD
HDIDLGDSRVVIQDYDNQHE
COD
S
M
P
F
SSC-A
FSC-A
68.8%
FSC-H
FSC-W
99.2%
SSC-A
Live/dead
97.3%
SSC-A
CD19
98.7%
IgG
IgA
33.6%
CD27
CD21
AM
7.8%
RM
83.5%
TLM
2.5%
IM
6.2%
G
IgG
IgA
CD21
CD27
HEVCA
IgG
IgA
IM
AM
TLM
RM
HEVCA
t-SNE2
t-SNE1
H
IgG
IgA
100
50
0
% CS mB
RM
AM
TLM
IM
RM
AM
TLM
IM

Fig. S1. HEV-CA reactivity of serum IgG antibodies and memory B cells from HEV seroconverters. (**A**) Sequence and structure diagrams of the HEV-CA protein monomer (PDB ID: 2ZTN) with individual domains highlighted in color. The surface representation of HEV VLP is shown on the bottom right. (**B**) SDS-PAGE and immunoblot analyses of purified recombinant HEV-CA (ORF2126-601) protein. H, heated: NH, non-heated. (**C**) ELISA graphs showing the reactivity of purified serum IgG antibodies from HEV-exposed donors against recombinant CA proteins covering different regions/domains. Black line corresponds to purified serum IgGs from a non-exposed donor used as negative control (Ctr$^-$). (**D**) Competition ELISA for binding to P or SM proteins with purified serum IgG antibodies in the presence of PM protein as a potential competitor. Blocking percentages are presented in the heatmap on the bottom right, with darker color indicating stronger binding inhibition. (**E**) Bar graph comparing the ELISA reactivity of serum IgG antibodies purified from HEV-exposed donors to HEV-CA overlapping peptides. Each bar presents the cumulative signal for all donors against a single peptide determined in two independent experiments. The white line represents the reactivity of purified IgG antibodies from HEV-seronegative donors (n=3) (65), used as negative controls, against HEV-CA overlapping peptides. Error bars indicate the SD. (**F**) Flow cytograms showing the gating strategy used to identify circulating blood HEV-CA-reactive IgG+ and IgA+ memory B cells prior to single cell sorting. (**G**) t-SNE-based analysis comparing the distribution of HEV-CA-binding B cells among the different memory lymphocyte phenotypes defined by CD21 and CD27 surface markers (CD27$^-$CD21$^+$ IM, intermediate memory; CD27$^+$CD21$^-$ AM, activated memory; CD27$^+$CD21$^+$ RM, resting memory; CD27$^-$CD21$^-$ TLM, tissue-like memory). (**H**) Dot plots showing the percentage of HEV-CA-reactive IgG$^+$ and IgA$^+$ memory B cells according with their phenotype as defined in (B).

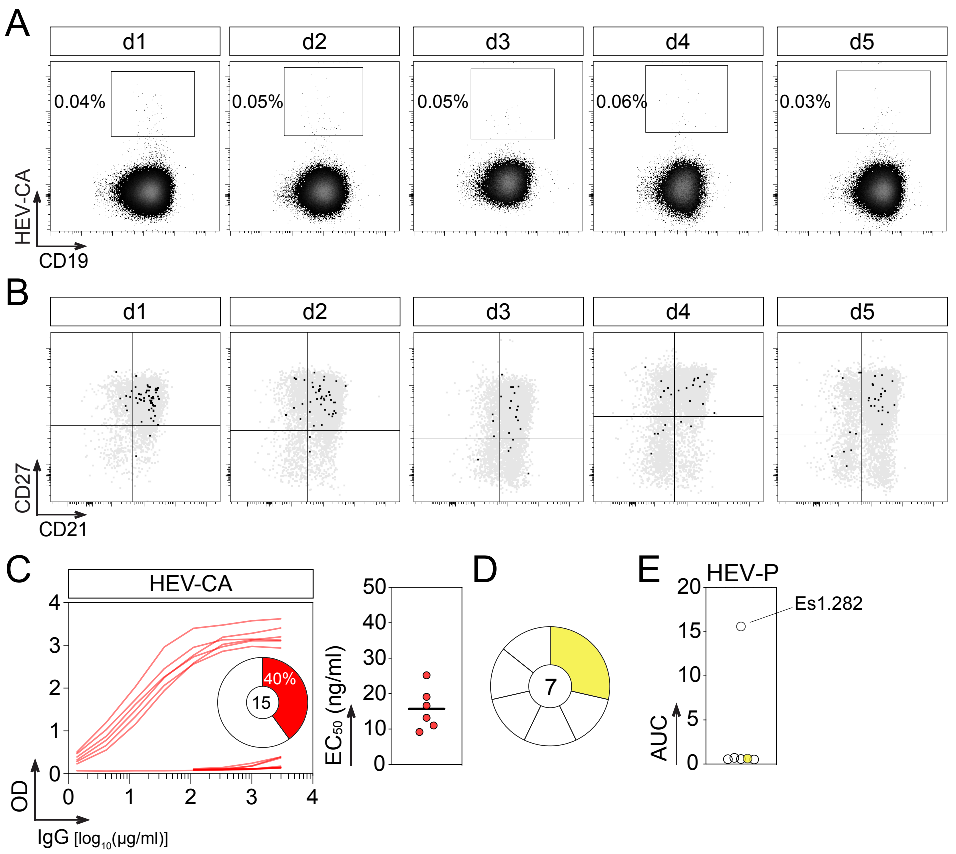

Fig. S2. Human HEV IgA memory B-cell antibodies. (**A**) Flow-cytometric plots showing the percentage of circulating blood HEV-CA-binding IgA⁺ B cells (gated on alive IgA⁺ lymphocyte singlets) from HEV-exposed individuals. (**B**) Flow cytometric plots showing the distribution of HEV-CA⁺ IgA⁺ memory B cells according to the expression CD27 and CD21 surface markers. (**C**) ELISA graph (left) showing the reactivity of IgA memory B-cell antibodies against purified recombinant HEV-CA protein. The pie chart indicates the proportion of CA-specific IgAs among total cloned antibodies. Dot plot shows their EC50 values for in vitro HEV neutralization (right). (**D**) Pie chart showing the distribution of clonally expanded (yellow) vs unique (white) HEV-CA IgA+ B-cell clones, with the slide size being proportional to the number of clonal members. The total number of expressed antibodies is indicated in the center. (**E**) Dot plot showing the reactivity of IgA memory B-cell antibodies against the P domain.

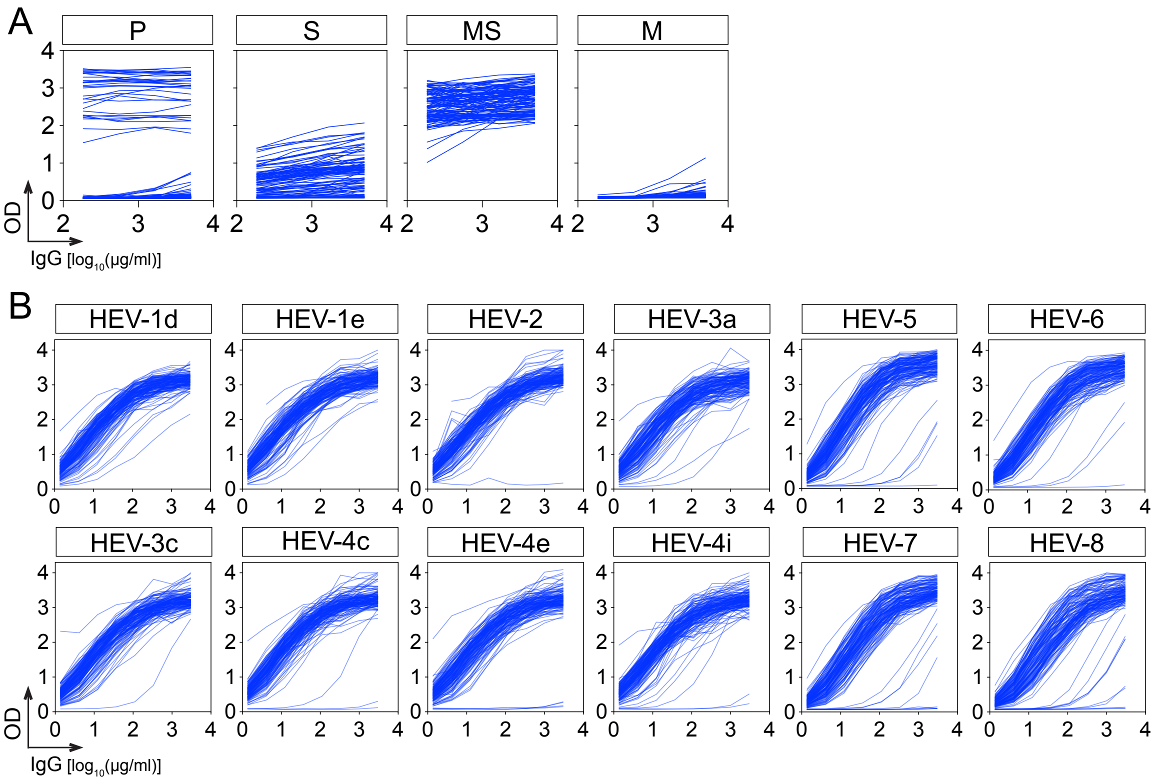

Fig. S3. Reactivity of human HEV memory B-cell antibodies. (**A**) Representative ELISA graphs showing the reactivity of HEV-CA-specific antibodies against purified recombinant proteins covering different HEV-CA domains. (**B**) Same as in (A) but for entire HEV-CA proteins of different *Paslahepevirus* HEV genotypes.

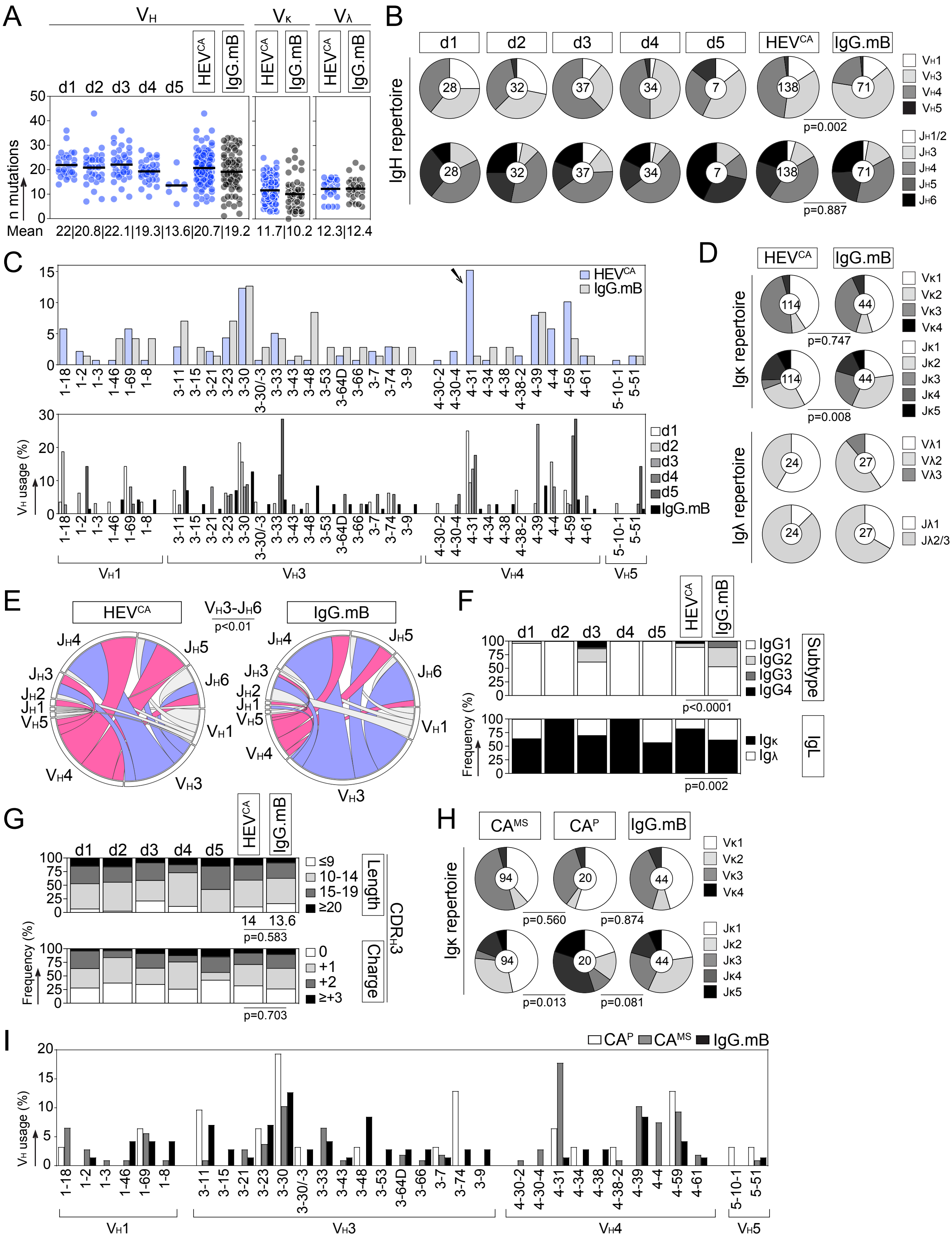

A
VH
Vκ
Vλ
d1 d2 d3 d4 d5
HEVCA
IgG.mB
n mutations
Mean
22|20.8|22.1|19.3|13.6|20.7|19.2
11.7|10.2
12.3|12.4
B
IgH repertoire
d1 d2 d3 d4 d5 HEVCA IgG.mB
28 32 37 34 7 138 71
p=0.002
p=0.887
VH1 VH3 VH4 VH5
JH1/2 JH3 JH4 JH5 JH6
C
HEVCA
IgG.mB
VH usage (%)
d1 d2 d3 d4 d5 IgG.mB
1-18 1-2 1-3 1-46 1-69 1-8 3-11 3-15 3-21 3-23 3-30 3-30/-3 3-33 3-43 3-48 3-53 3-64D 3-66 3-7 3-74 3-9 4-30-2 4-30-4 4-31 4-34 4-38 4-38-2 4-39 4-4 4-59 4-61 5-10-1 5-51
VH1 VH3 VH4 VH5
D
HEVCA IgG.mB
Igκ repertoire
114 44
p=0.747
p=0.008
Vκ1 Vκ2 Vκ3 Vκ4
Jκ1 Jκ2 Jκ3 Jκ4 Jκ5
Igλ repertoire
24 27
Vλ1 Vλ2 Vλ3
Jλ1 Jλ2/3
E
HEVCA
VH3-JH6
p<0.01
IgG.mB
JH1 JH2 JH3 JH4 JH5 JH6 VH1 VH3 VH4 VH5
F
d1 d2 d3 d4 d5 HEVCA IgG.mB
Frequency (%)
IgG1 IgG2 IgG3 IgG4
Subtype
p<0.0001
Igκ Igλ
IgL
p=0.002
G
d1 d2 d3 d4 d5 HEVCA IgG.mB
Frequency (%)
≤9 10-14 15-19 ≥20
Length
14 13.6
p=0.583
0 +1 +2 ≥+3
Charge
p=0.703
CDRH3
H
CAMS CAP IgG.mB
Igκ repertoire
94 20 44
p=0.560 p=0.874
p=0.013 p=0.081
Vκ1 Vκ2 Vκ3 Vκ4
Jκ1 Jκ2 Jκ3 Jκ4 Jκ5
I
CAP CAMS IgG.mB
VH usage (%)
VH1 VH3 VH4 VH5

Fig. S4. Immunoglobulin gene repertoire of human HEV IgG⁺ memory B cells. (**A**) Dot plots comparing the number of somatic mutations in V_H, Vκ and Vλ genes of HEV-CA (HEV-CA, blue) and control (IgG.mB, black) IgG⁺ memory B-cell antibodies. Averaged mutation per donor and group is indicated below. (**B**) Pie charts comparing the distribution of V_H and J_H gene usage of HEV-CA and control (IgG.mB) IgG⁺ memory B-cell antibodies. The number of antibody sequences analyzed is indicated in the center of each pie chart. Groups were compared using 2 × 5 Fisher's Exact test. (**C**) Bar graph (top) comparing the distribution of single V_H genes expressed by total HEV-CA and control (IgG.mB) IgG⁺ memory B-cell antibodies (as frequencies of total) (top) and for each HEV-expressed donor (bottom). (**D**) Same as in (B) but for total Vκ / Jκ and Vλ / Jλ gene usages. (**E**) Circos plot comparing the V_H-D_H-J_H rearrangement frequencies between HEV-CA and control (IgG.mB) IgG⁺ memory B-cell antibodies. (**F**) Bar graphs comparing the distribution of IgG subclass (top) and κ- vs λ-Ig chain usage (bottom) between HEV-CA and control (IgG.mB) IgG⁺ memory B-cell antibodies. Groups were compared using 2 × 5 Fisher's Exact test. (**G**) Same as in (F) but for amino acid length and total positive charges of the CDR_H3. (**H**) Pie charts comparing the distribution of Vκ / Jκ gene usages between anti-CA P domain (CA^P) and anti-CA MS domains (CA^{MS}) IgG antibodies. Groups were compared using 2 × 2 Fisher's Exact test. (**I**) Bar graph comparing the distribution of single V_H genes by anti-CA P domain (CA^P), anti-CA MS domains (CA^{MS}) and control (IgG.mB) IgG⁺ memory B-cell antibodies (as frequencies of total).

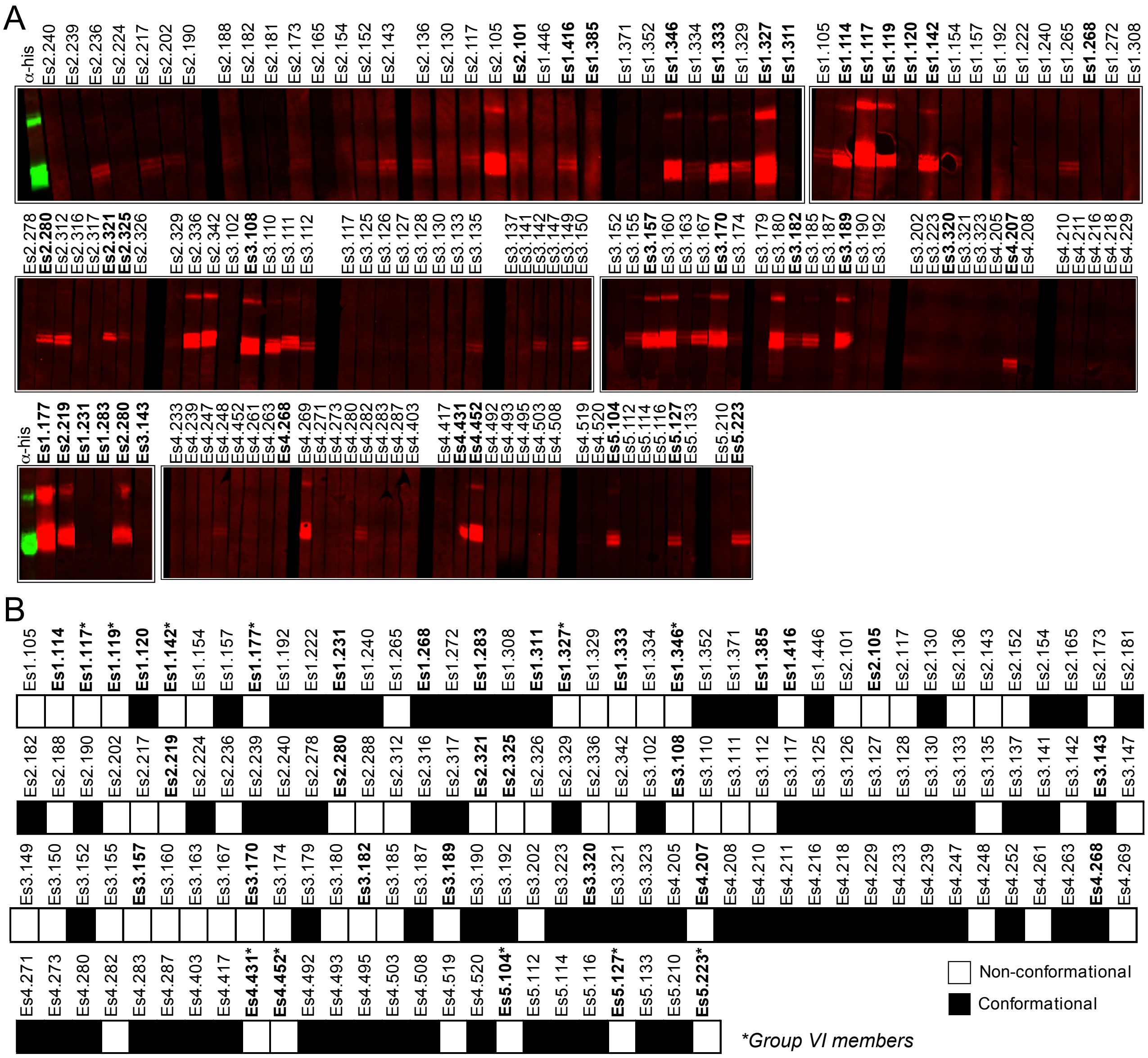

Fig. S5. Immunoblotting reactivity of human HEV antibodies to denatured CA protein. (**A**) Infrared immunoblot showing the reactivity of HEV IgG antibodies (n=138) to denatured HEV-CA protein. Immunoreactive green bands correspond to the denatured protein probed with an anti-6xHis tag antibody. Immunoreactive red bands revealed the binding of human HEV antibodies to denatured HEV-CA. (**B**) Table showing the categorization of HEV antibodies as binding conformational (black) or non-conformational (white) epitopes based on immunoblotting shown in (A). Anti-P antibodies are highlighted in bold, and group VI members marked with an asterisk.

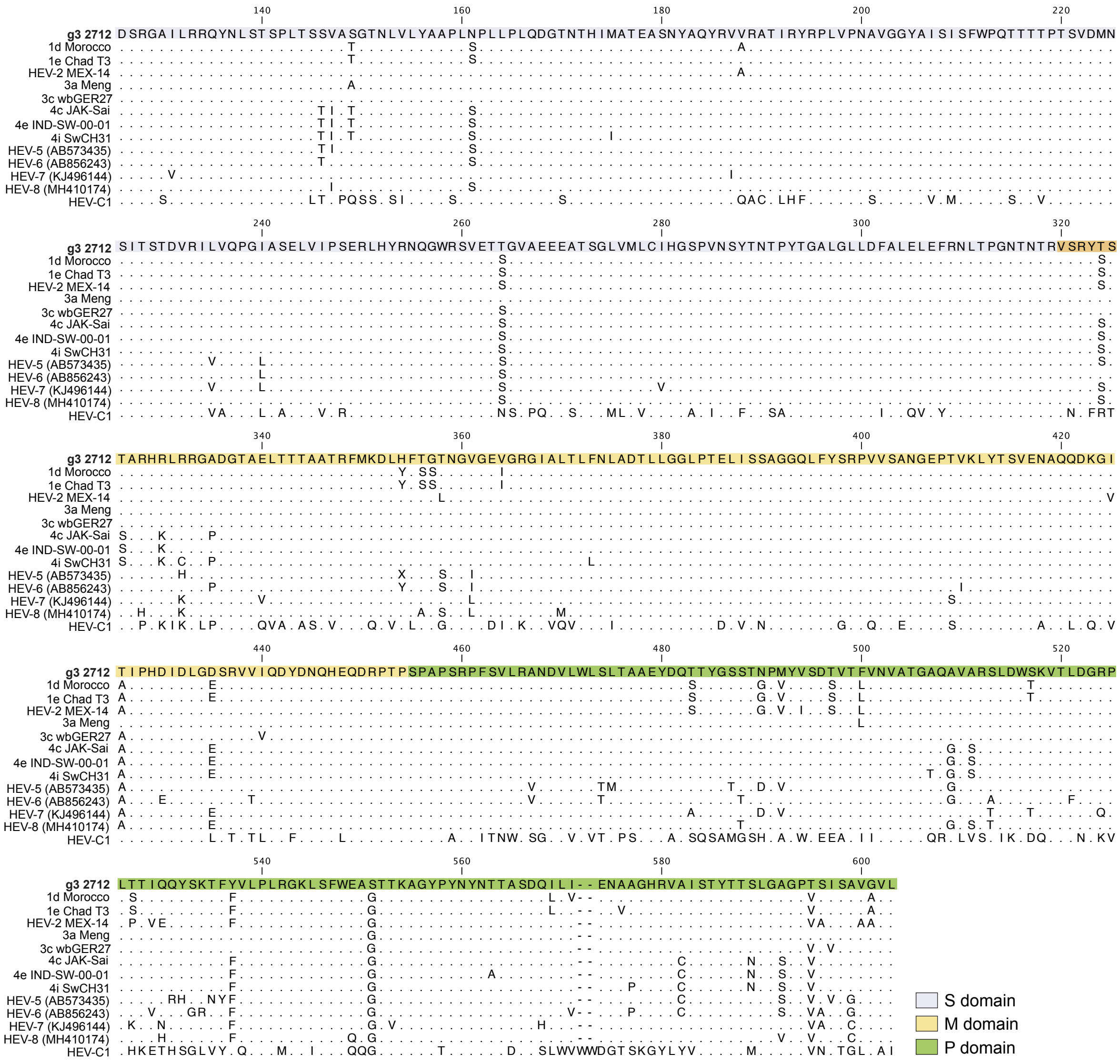

Fig. S6. *Paslahepevirus* and *Rocahepevirus* capsid proteins. Amino acid alignment of the selected *Paslahepevirus* and *Rocahepevirus* capsid sequences as compared to the HEV g3-2712 genotype with the shell (S), middle (M) and protruding (P) domains colored in grey, yellow and green, respectively.

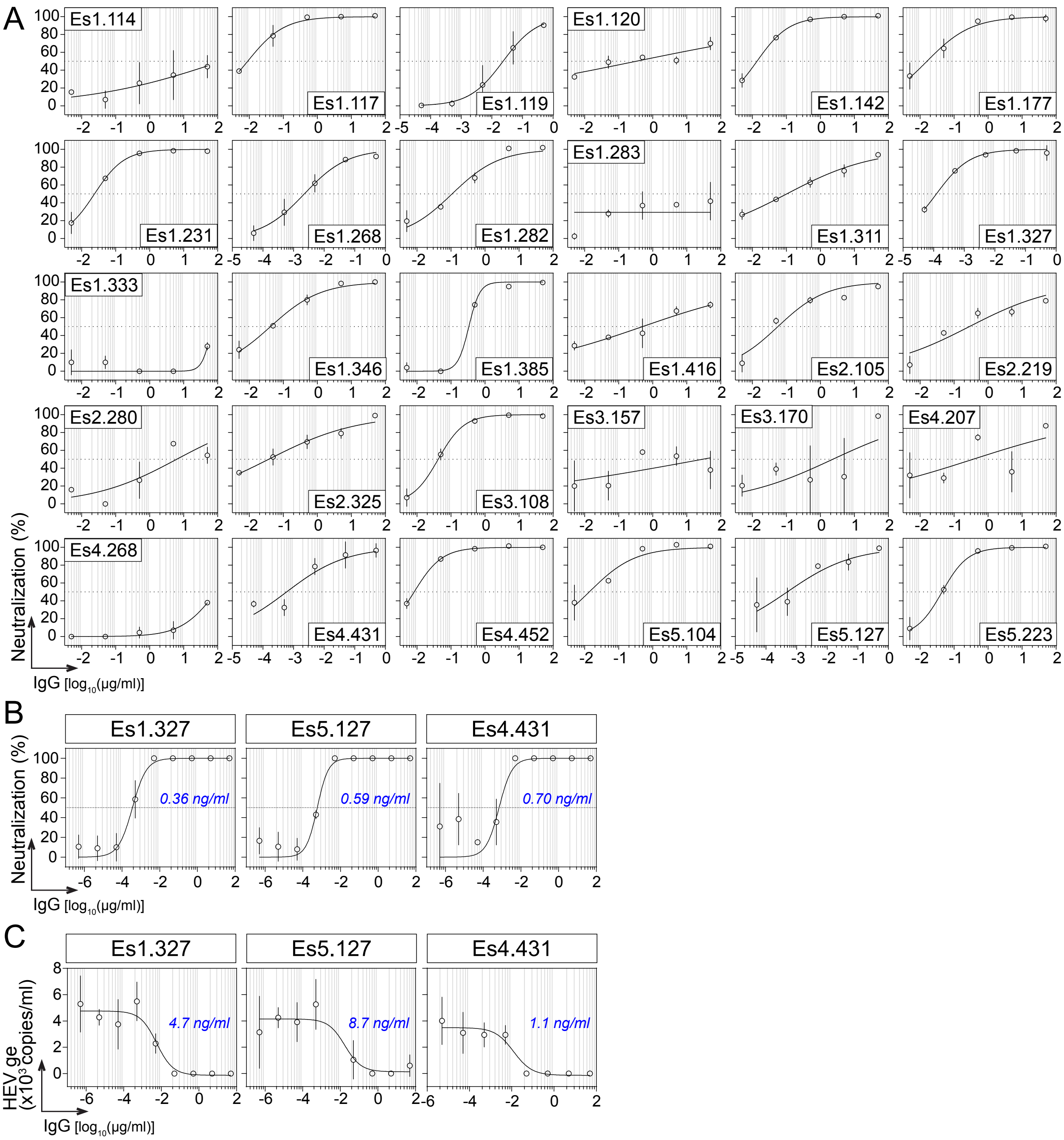

Fig. S7. HEV neutralizing activity of human anti-CA monoclonal antibodies. (**A**) Graph showing the neutralization curves of real naked genotype 3 HEV by human ant-CA IgG antibodies as measured in vitro using HepG2/C3A MAVS cells as targets. The dotted horizontal line indicates 50% of neutralization. (**B**) Neutralization curves as in (A) but with a broader concentration range for highly potent antibodies Es1.327, Es4.431 and Es5.127. (**C**) Same as in (B) but quantifying HEV RNA copy numbers in culture supernatants. IC_{50} values are indicated in blue. Error bars in (A-C) indicate the SEM of duplicate values and are representative of at least two independent determinations.

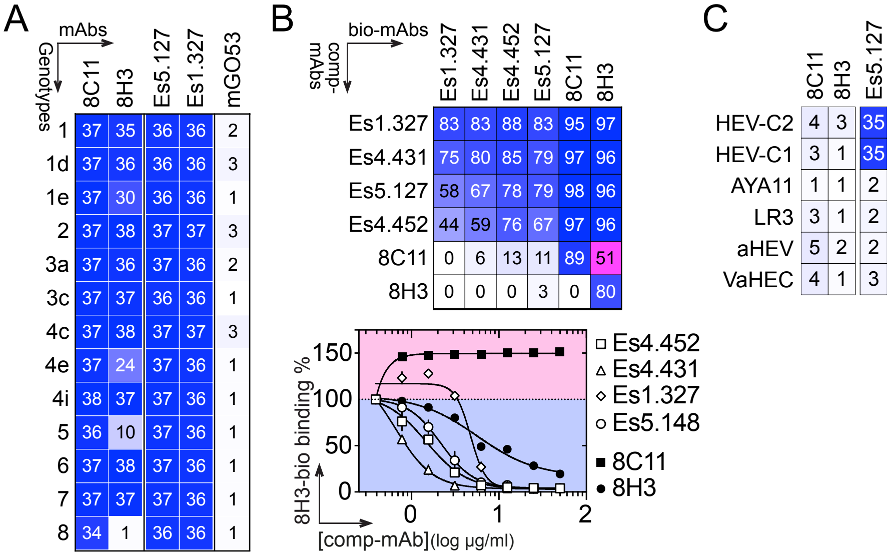

Fig. S8. Binding comparison of potent human HEV neutralizing antibodies with murine antibodies 8C11 and 8H3. (**A**) Heatmap comparing the ELISA reactivity of selected murine (8C11 and 8H3) and human (Es5.127 and Es1.327) anti-P monoclonal IgG antibodies (mAbs) against purified HEV-CA proteins from different genotypes measured as area under the curve (AUC) values with serially-diluted IgGs. Darker- and white-colored cells indicate high and no or low binding, respectively. Means of intra-assay duplicates are shown. (**B**) Competition ELISA heatmap (top) showing the level of binding inhibition to HEV-CA by biotinylated anti-P antibodies (bio-mAbs) in the presence of potential antibody competitor (comp-mAbs). Darker blue and white colored cells indicate high and no or low inhibition, respectively. Pink colored cell indicates enhanced binding. Means of intra-assay duplicates are shown. Representative competition ELISA graph comparing the binding profile of biotinylated 8H3 (-bio**)** in the presence of selected potential anti-P antibody competitors. Means ± SD of duplicate values are shown. (**C**) Heatmap showing the binding assessment of murine anti-P antibodies 8C11 and 8H3 against purified recombinant P proteins from selected *Orthohepevirinae* genera (*Rocahepevirus*, *Chirohepevirus* and *Avihepevirus*). The mean AUCs from duplicate titration values are shown, and the binding profile of Es5.127 tested in parallel (Fig.3F) is displayed for comparison.

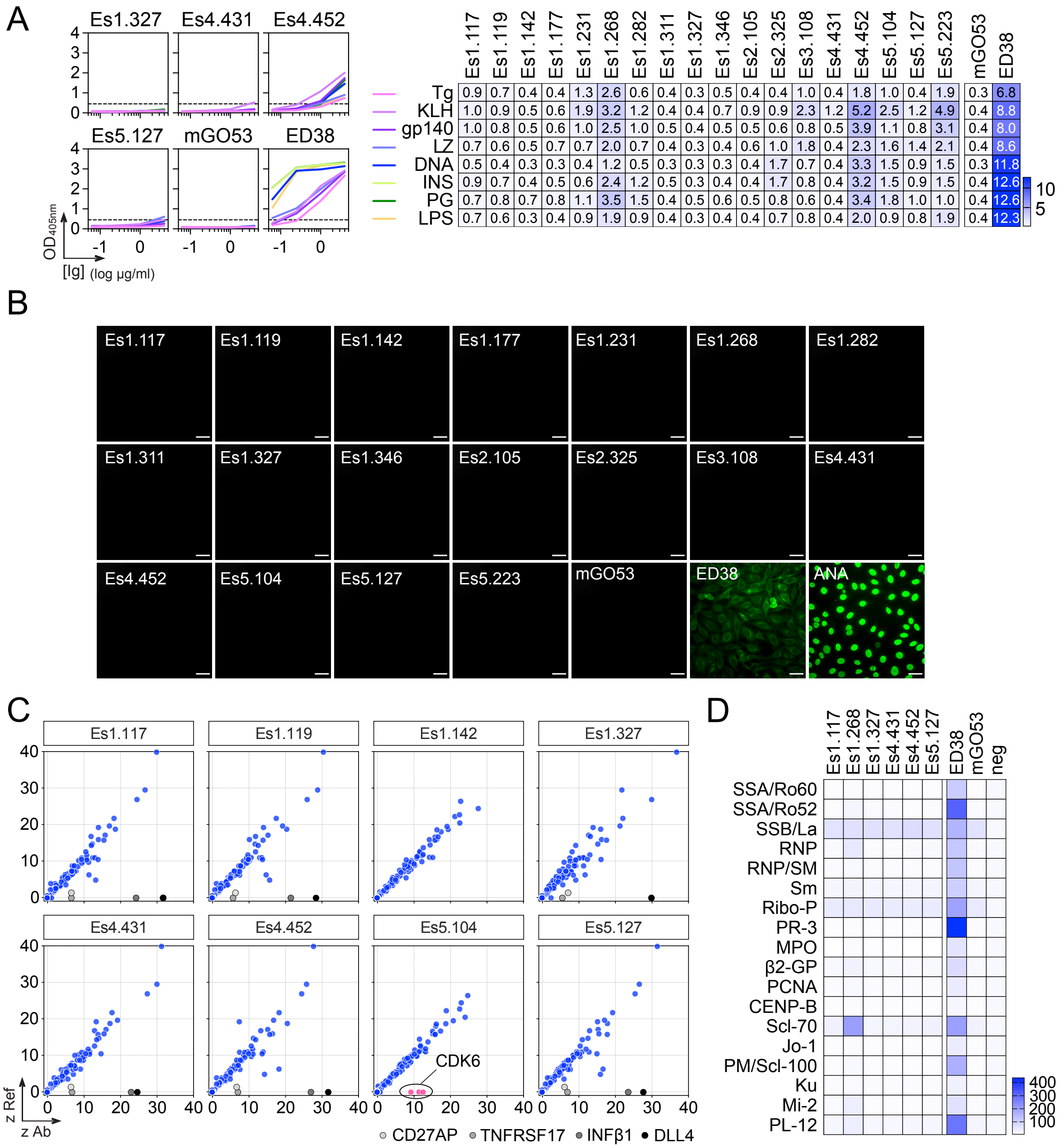

Fig. S9. Off-target binding characteristics of potent HEV neutralizing antibodies. (**A**) Heatmap showing the ELISA reactivity of selected HEV neutralizing IgG antibodies against a panel of structurally unrelated antigens. Polyreactive (ED38) and non-polyreactive (mGO53) controls are included. Color value is proportional to the reactivity level measured as the area under the curve (AUC). (**B**) Microscopic images showing the reactivity of selected HEV neutralizing IgG antibodies to HEp2-expressing self-antigens assayed by indirect immunofluorescence assay. The negative (mGO53), low-positive (ED38), and kit's positive (ANA) controls are included. The scale bars represent 40 µm. (**C**) Microarray plots showing the reactivity selected HEV neutralizing IgG antibodies to human proteins. Each spot corresponds to the z-scores given on a single protein by the reference antibody (z Ref: mGO53, y axis) and test antibody (x axis). (**D**) Heatmap comparing the reactivity profiles of selected HEV neutralizing IgG antibodies against a panel of self-antigens as measured by a multiplex bead-based binding assay. ED38 (polyreactive), mGO53 (non-polyreactive) and kit's negative (neg) controls antibodies are included. Color values are proportional to the median fluorescent intensity (MFI) of mean duplicates.

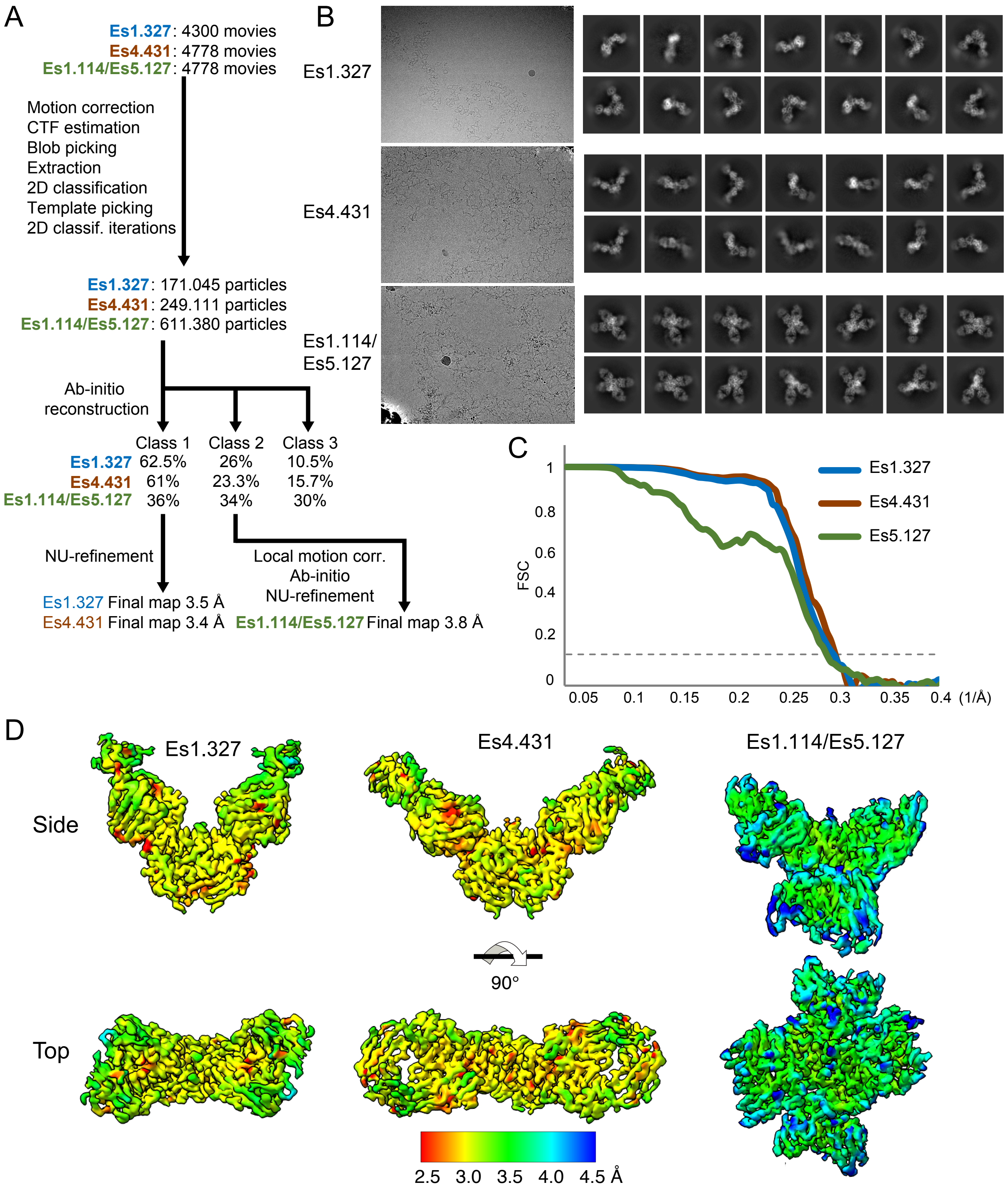

Fig. S10. Cryo-EM data processing of HEV-CA protein in complex with Es1.327, Es4.431 and Es5.127 Fabs. (**A**) Workflow for cryo-EM data processing performed with cryoSPARC. (**B**) Representative electron micrograph and 2D-classes for each complex. (**C**) Fourier-shell correlation (FSC) as a function of the spatial frequency indicating estimated resolutions for the final reconstructions. (**D**) Overall maps of the complexes colored by local resolution.

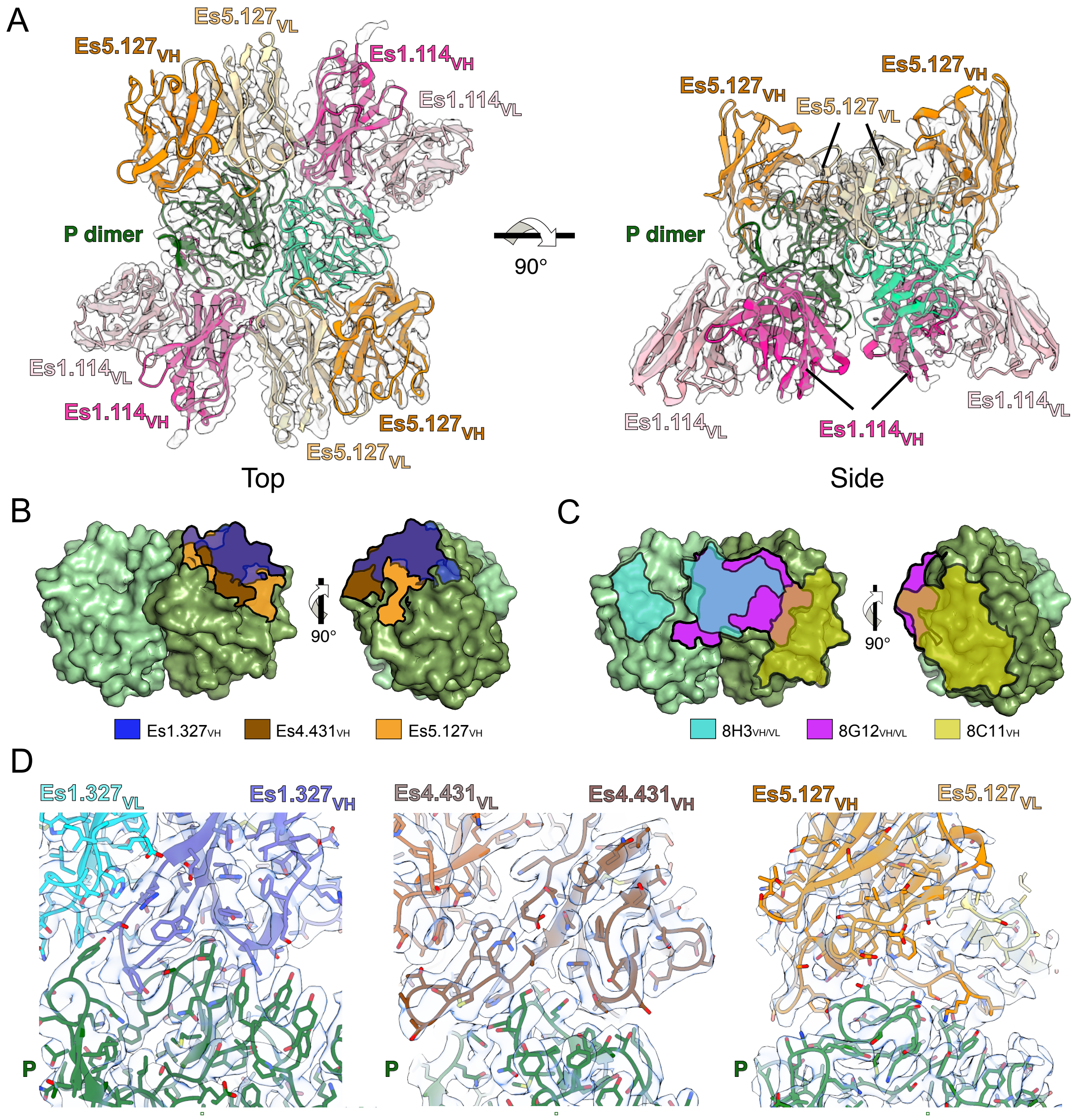

Fig. S11. Models of protein fitting into electron density maps. (**A**) Ribbon model of the ternary complex of *Paslahepevirus* HEV capsid with Fabs Es5.127 and Es1.114 fitted into the overall map of the complex. (**B**) Binding surface representation of the interaction zones of Fabs Es1.327, Es4.431 and Es5.127. (**C**) Same as in (B) but for murine antibodies 8C11 (PDB ID: 6LB0), 8G12 (PDB ID: 4PLK) and 8H3 (PDB ID: 9IY2). P domain is colored in shades of green. (**D**) close-up views of the interaction of P domain with the VH chain of Fabs Es1.327, Es4.431 and Es5.127.

Table S1. Characteristics of HEV-exposed immune donors

					anti-CA serum IgG/IgA			CA-reactive B cells (%)		n HEV mAbs	
	**Age**	**Gender**	**Chronic HCV**	**HEV IgG titers***	**IgG EC_{50}** (µg/ml)	**IgA EC_{50}** (µg/ml)	**IC_{50}** serum IgG	**IgG**	**IgA**	**IgG**	**IgA**
**d1**	55	M	yes	20.07	5.33	NC	89	0.1	0.04	28	6
**d2**	53	M	yes	2.72	53.68	NC	>	0.04	0.05	32	/
**d3**	42	M	yes	14.16	11.65	NC	48	0.2	0.05	37	/
**d4**	56	F	yes	19.43	2.43	NC	38	0.22	0.06	34	/
**d5**	61	M	yes	2.55	45.04	NC	>	0.03	0.03	7	/

F, female; M, male. *EIA Wantai values > 1 are considered positive. > indicates that IC50 values > 200 µg/ml.
NC, EC_{50} values could not be calculated.

Table S2. Immunoglobulin gene repertoire, reactivity and antiviral activity of human HEV memory B-cell antibodies.

mAb	Donor	IgH V_H	D_H	J_H	Subtype	CDR_H3 Sequence (aa)	Length	(+)	(-)	V_H MUT	SHM (%)	IgL	V_L	J_L	CDR_L3 Sequence (aa)	Length	(+)	(-)	V_L MUT	SHM (%)	Clonotype members (N)	Reactivity HEV g3-2712 (AUC) HEV-CA	P	PM	M	MS	S	Reactivity HEV-CA, Paslahepevirus HEV genotype (EC_{50} ng/ml) 1d	1e	MEX-14	3a	3c	4c	4e	4i	5	6	7	8	Rocahepevirus HEV-C1 (AUC)	Neutralization (%)	Neutralization (IC50 µg/ml)
Es1.105	d1	4-31	3-22	3	γ1	EGDSSGFGSGYYPRGVFDI	19	1	3	19	5	κ	1D-39/1-39	3	QQNYRTPIFT	10	1	0	6	2	1	16	0.4	0.4	0.5	11	4.5	7.4	5.6	5.9	8.9	7.8	9.5	7.4	6.7	15.3	17.5	16.1	13.5	5.2	26	ND
Es1.114	d1	3-11	3-3	4	γ1	EPTYHDFGSGYYTGFDY	17	1	3	17	5	κ	1D-39/1-39	3	QQSYNLFT	8	0	0	9	3	3	15	15	16	ND	0.9	ND	8.6	10.1	5.1	7.2	5.5	9.8	7.6	4.9	9.0	13.4	NS	13.4	0.4	44	50
Es1.117	d1	4-31	3-10/4-11	4	γ1	RRDGTIDY	8	2	2	27	8	λ	2-11	3/2	CSYAGAYTLGV	11	0	0	16	5	2	16	15	16	ND	0.3	ND	8.5	8.9	4.3	6.4	5.5	9.2	7.7	4.9	10.7	13.3	14.0	16.4	0.2	100	0.0089
Es1.119	d1	3-30/3-30-3	2-2	5/4	γ1	DQLSDIVPVPAAPYLRN	17	1	2	24	7	λ	1-51	3	GTWDSSLNSWV	11	0	1	14	5	4	16	15	16	ND	0.3	ND	7.3	8.0	5.0	7.8	7.0	8.8	8.7	5.9	8.4	8.5	9.8	88.6	5.5	100	0.0145
Es1.120	d1	4-59	3-10	6	γ1	DKAGNMPARPAPPYNYGLDV	20	2	2	19	5	κ	1-5	1	QQYNSYSPWT	10	0	0	8	3	5	16	17	16	ND	0.4	ND	13.9	14.3	7.4	13.8	13.8	15.2	10.7	9.5	11.0	17.1	15.6	17.7	0.3	70	0.3226
Es1.142	d1	3-30/3-30-3	2-15/3-10/2-21	5	γ1	VRGHCPATSCYGDS	14	2	1	23	7	λ	1-51	3	GTWDSSLSAWV	11	0	1	9	3	2	15	15	16	ND	0.4	ND	9.9	9.0	7.0	7.2	14.7	11.2	8.2	13.6	7.1	10.0	9.6	444.6	5.5	100	0.0137
Es1.154	d1	4-31	3-10/3-16	5	γ1	ESVMIRGLHIWFDP	14	2	2	20	6	κ	1-5	2	QQYNSYSPYT	10	0	0	20	7	5	15	0.4	0.4	0.5	12	4.8	10.3	8.4	7.8	14.0	10.0	9.8	10.9	9.1	17.1	20.9	21.7	16.5	4.6	43	ND
Es1.157	d1	3-30	3-9	5	γ1	IRAFDWSLMGWFDP	14	1	2	23	7	κ	1-12	4	QQANSFPLT	9	0	0	16	6	1	15	2	1.8	1.5	11	0.8	9.5	10.4	7.8	13.1	9.7	6.9	14.3	12.0	14.3	19.8	24.9	24.2	3.8	11	ND
Es1.177	d1	4-38-2	3-9	5	γ1	FLRYQSIDS	9	1	1	26	8	λ	1-44	3	ATWEDSLNGRV	11	1	2	15	5	1	16	16	15	ND	0.4	ND	10.3	9.2	6.3	7.7	8.6	9.6	6.4	9.2	7.1	9.1	9.5	11.6	0.1	98	0.0160
Es1.192	d1	1-46	4-11/3-3	3	γ2	DPPLYSFGQNDAFDI	15	0	3	25	7	κ	3-20	1	QQYGSSPRT	9	1	0	17	6	3	15	0.4	0.4	0.4	10	2.1	16.9	20.7	18.9	48.3	16.3	18.5	20.4	20.2	12.9	17.1	26.7	17.9	0.1	10	ND
Es1.195	d1	4-31	3-10	6	α1	DWGSGTYYDGHIYYGMDV	18	1	3	13	4	κ	3-15	2	QQYHNWPLYT	10	1	0	20	2	1	16	0.6	0.5	ND	15	0.6	6.0	9.1	8.6	7.4	5.9	8.7	6.0	12.4	12.0	13.3	14.6	16.4	4.9	ND	ND
Es1.222	d1	4-31	1-26	5	γ1	GGTWEGWLDP	10	0	2	33	10	κ	3-15	1	QQSYNWPRT	9	1	0	4	1	2	17	0.5	0.4	ND	16	1.6	9.5	11.3	15.3	6.3	9.1	8.2	11.2	9.7	10.2	10.2	12.0	10.1	4.4	25	ND
Es1.231	d1	3-30	4-17	5	γ1	SAYGDGGGWFDP	12	0	2	18	6	λ	2-14	3/2	SSYTTSRTVV	10	1	0	17	6	26	17	16	15	ND	0.5	ND	12.9	9.6	8.9	10.4	13.8	15.1	NS	11.4	36.5	26.6	21.6	NS	3.1	98	0.0239
Es1.237	d1	4-31	5-12	4	α1	GAEWLRFFDS	10	1	2	11	3	κ	3-20	1	QHYDNLWWT	9	1	1	26	5	1	18	0.6	0.6	ND	14	0.5	9.4	6.2	7.4	5.4	6.1	5.1	7.1	6.8	23.6	17.5	30.4	18.5	ND	ND	ND
Es1.240	d1	1-69/1-69D	3-16	3	γ1	FGRRVEDSDAFDI	13	2	4	25	8	κ	2D-28/2-28	2	MQALQTPYT	9	0	0	11	4	5	16	0	0.4	0.6	13	1.2	12.1	14.4	14.1	11.3	13.9	15.9	14.2	11.2	ND	ND	ND	ND	ND	31	ND
Es1.243	d1	3-48	3-16	4	α1	GRHDNVWGSSPFDY	14	2	2	11	3	κ	4-1	4	QQYYTVPLT	9	0	0	23	4	1	16	0.7	0.6	ND	15	0.4	7.1	8.8	6.6	6.6	8.2	8.9	10.1	11.5	12.3	14.1	17.2	22.7	5.1	ND	ND
Es1.265	d1	4-31	6-19	5	γ1	GHNSGLNNWFDP	12	1	1	17	5	κ	1D-39/1-39	2	QQSYNIPYT	9	0	0	10	4	1	15	0.4	0.4	1	11	5.5	9.8	9.2	13.3	12.7	8.0	9.3	10.5	6.7	31.3	35.1	34.9	37.5	4.5	14	ND
Es1.268	d1	3-30	3-10	4	γ1	DRAVYYGLGWGSADN	15	1	2	25	7	λ	2-14	3	SSHTIKSTWV	10	2	0	16	5	7	10	9.3	14	ND	0.4	ND	8.2	12.4	10.4	7.6	14.3	9.4	7.2	7.5	115.4	18.6	NS	20.5	0.2	100	0.031
Es1.272	d1	4-31	5-5/5-18	4	γ1	EHSFGHEVYFDQ	12	2	3	19	6	κ	1-5	3	QQYNSYSRA	9	1	0	7	3	1	16	1	1.2	0.6	10	0.4	13.5	20.3	18.0	20.7	18.0	16.0	17.6	17.0	32.3	38.1	50.1	48.4	4.2	16	ND
Es1.282	d1	4-61	5-5/5-18/3-10	4	α1	DAGRGRVYGHFDH	13	4	2	36	11	κ	1D-39/1-39	3	QQSYSTPFT	9	0	0	32	9	1	16	16	16	ND	0.7	ND	10.2	8.6	NS	4.7	5.3	14.7	15.2	7.6	NS	23.7	NS	NS	0.2	100	0.11
Es1.283	d1	4-59	2-8	4	γ1	EGFERPSGLRYFDNNGGGYYFDY	23	2	4	14	4	κ	3-11	5	QQRGNWPPVT	10	1	0	5	2	4	17	16	15	ND	1.3	ND	10.7	11.0	12.1	10.2	35.5	16.9	13.3	15.4	21.9	34.4	13.3	26.1	0.5	42	50
Es1.308	d1	4-38-2	1-26/3-16	4	γ1	DWVKWGDYFDY	11	1	3	19	6	λ	2-11	1	SSYAGTYT	8	0	0	12	4	6	15	0.4	0.4	0.5	10	3.7	11.2	28.8	16.1	18.8	17.6	13.1	15.6	11.4	25.5	19.9	49.6	19.1	4.3	20	ND
Es1.311	d1	3-30	3-3/3-9	4	γ1	DRILGHFEWFPGDFDY	16	2	4	21	6	κ	3-11	4	QQRSNWPLT	9	1	0	10	4	3	16	15	15	ND	0.3	ND	6.2	5.9	6.2	5.1	10.0	6.3	11.6	7.8	6.7	8.4	9.7	10.3	0.1	94	0.1018
Es1.316	d1	1-69	3-16/3-9	4	α1	DWRLGPSFEN	10	1	2	23	7	κ	2D-28/2-28	2	MQALQPPYT	9	0	0	3	1	1	15	0.6	0.6	ND	12	0.4	6.7	8.8	13.3	13.0	14.2	8.3	15.9	9.3	15.0	23.3	16.2	19.5	0.1	ND	ND
Es1.327	d1	1-18	1-14	4	γ1	VWTGYGTNRGDY	12	1	1	36	11	κ	1D-39/1-39	4	QQSDNNFALT	10	0	1	23	8	2	16	15	15	ND	0.5	ND	9.1	7.2	3.7	6.4	8.0	9.7	9.2	6.5	6.9	7.9	9.9	10.3	0.1	100	0.0004
Es1.329	d1	3-11	6-19	3	γ1	TVGSGWTLDASDV	13	0	2	27	8	κ	3-15	1	QQYNNWPGT	9	0	0	6	2	1	17	0.4	0.4	0.4	11	4.9	7.6	13.4	10.2	11.0	10.5	7.4	11.4	7.8	13.5	14.3	16.8	13.8	4.9	11	ND
Es1.333	d1	1-69/1-69D	3-22	4	γ1	SNTYYYDTSGYPRHKEGGRYFDY	23	4	3	22	6	λ	1-44	3	AAWDDSMNGLWV	12	0	2	5	2	4	16	16	15	ND	ND	ND	14.0	10.6	19.8	9.4	16.3	13.7	15.6	14.0	10.1	12.4	15.2	15.2	0.1	28	50
Es1.334	d1	4-31	3-16	5	γ1	DLGNWIYAGFDP	12	0	2	20	6	κ	3-15	2	QQYYRWPLYT	10	1	0	9	3	4	15	0.6	0.8	ND	13	2	9.9	10.8	16.6	10.7	9.4	7.4	11.5	8.0	43.9	44.2	56.6	45.7	ND	ND	ND
Es1.346	d1	3-7	2-2	4	γ1	DRGAIVPVPTAIYSDY	16	1	2	19	6	λ	1-51	3/2	GTWDTGLSAVV	11	0	1	9	3	1	17	15	15	ND	0.5	ND	5.1	5.4	6.4	5.6	6.0	6.8	8.2	5.8	10.9	16.5	13.4	953.2	5.6	100	0.042
Es1.352	d1	1-69/1-69D	5-18/3-10/3-16	4	γ1	DTAYGIYAGY	10	0	1	27	8	κ	2D-28/2-28	1	MQALQTPRT	9	1	0	3	1	4	17	0.4	0.4	ND	15	1.8	15.5	11.1	15.2	13.3	11.2	15.3	18.8	14.5	10.7	11.8	14.7	14.7	0.2	37	ND
Es1.371	d1	1-8	6-19	6	γ1	GGAAGSSGWYVENNYYTMDV	20	0	2	16	5	κ	1-5	2	QQYNTFPYT	9	0	0	12	5	2	15	0.4	0.4	0.4	10	4.1	11.0	10.5	12.9	9.7	9.4	8.1	13.1	11.9	31.6	16.2	54.6	15.8	0.1	15	ND
Es1.385	d1	3-30-3	6-19	3	γ1	AVTPLYNSGWYNGFDI	16	0	1	18	5	λ	2-14	3/2	SSYTPSNTRI	10	1	0	15	5	2	16	15	14	ND	0.4	ND	21.8	49.2	19.9	725.4	222.9	209.2	NS	72.5	NS	NS	NS	NS	0.2	100	0.43
Es1.387	d1	3-30-3	2-21/1-20/1-7	4	α1	VHELTADY	8	1	2	7	2	λ	2-8	3	NSYAGSNNLV	10	0	0	10	3	2	16	0.5	0.6	ND	15	0.5	6.7	8.1	7.7	5.7	7.9	6.3	7.4	5.2	9.5	12.2	11.9	13.0	4.7	ND	ND
Es1.416	d1	1-69	5-24	6	γ1	PLGDGYNSPLRRDNYGMDV	19	2	3	16	5	κ	1-33/1D-33	4	QQYNNLPLT	9	0	0	13	5	4	15	16	14	ND	0.4	ND	7.0	12.0	7.9	8.9	11.4	10.9	7.4	8.5	11.3	14.0	20.9	27.1	0.2	74	0.50
Es2.101	d2	3-30/3-33	5-5/5-18	4	γ1	DEGRGYTSVLDY	12	1	3	24	7	κ	3-11	4	QQRSNWPPG	9	1	0	10	4	1	19	0.3	0.4	0.4	11	4.8	7.8	7.4	7.4	7.8	5.6	5.4	8.1	8.9	13.9	13.9	19.1	16.0	4.6	32	ND
Es2.105	d2	3-74	2-21	4	γ1	DGIRCGGDCPYDY	13	1	3	8	2	κ	1-27	4	QKYNSAPLT	9	1	0	4	1	1	17	17	15	ND	ND	ND	5.4	15.9	13.5	6.5	9.7	6.4	7.0	8.9	9.6	10.9	11.0	13.4	0.1	95	0.056
Es2.117	d2	4-4	2-2/2-15	5	γ1	DIVAAGRGLNWFDP	14	1	2	26	8	κ	4-1	1	QQYYSFPPT	9	0	0	11	4	1	18	0.4	0.4	0.5	11	0.5	9.5	7.9	8.0	8.9	6.5	9.0	10.6	10.0	14.2	14.9	19.8	18.0	4.5	13	ND
Es2.130	d2	4-31	1-26	4	γ1	SRGTWELSLAY	11	1	1	24	7	κ	1-27	2	QSYNSALYT	9	0	0	9	3	2	19	0.5	0.5	0.9	10	2.2	9.5	10.0	10.9	11.3	9.0	14.2	17.4	14.6	54.3	25.8	76.3	44.4	2.9	11	ND
Es2.136	d2	1-18	5-24	6	γ1	DSGWGGIQLGGSFYYFGMDV	20	0	2	24	7	κ	3-20	1	QQYGVSPWT	9	0	0	14	5	1	18	0.8	0.4	0.8	11	4.2	11.7	10.1	13.4	21.3	10.0	10.1	15.2	17.2	13.9	14.2	21.1	22.4	4.4	17	ND
Es2.143	d2	1-18	3-22	6	γ1	VEYFYDSRGYPIPYAMDV	18	1	3	26	7	κ	4-1	1	QQYYGTPT	8	0	0	19	7	1	19	0.3	0.6	0.4	15	3.6	11.5	13.5	13.8	14.3	16.9	11.4	15.7	11.4	12.1	14.8	18.5	16.0	4.6	15	ND
Es2.152	d2	4-31	6-19	4	γ1	EAEWLGPFDS	10	0	3	29	9	κ	3-11	4	QQGSSWPRT	9	1	0	11	4	1	16	0.4	0.4	ND	16	0.4	16.0	18.0	20.0	17.7	27.1	17.1	18.7	18.6	15.3	14.5	19.7	14.9	4.7	17	ND
Es2.154	d2	3-23	4-23/4-17	4	γ1	DITDYVVYFDN	11	0	3	21	6	κ	3-15	1	QQYYNWPRT	9	1	0	12	4	3	18	0.3	0.5	0.5	9.4	3.5	89.8	51.5	24.6	227.7	83.0	NS	NS	NS	64.6	26.5	49.5	53.1	0.6	15	ND
Es2.165	d2	1-2	3-10/3-3	5	γ1	DRLALGVGEGAFGGFDP	17	1	3	22	6	κ	1-5	1	QQYNSYSPWT	10	0	0	7	3	1	19	0.3	0.5	0.5	11	1.1	21.6	17.1	22.5	32.5	32.0	35.4	22.4	18.8	46.1	28.6	89.0	39.6	3.4	1	ND
Es2.173	d2	4-30-2	2-8/5-24	2	γ1	GSQWPWYFDL	10	0	1	18	6	κ	3D-15	2	QQYYNWPPRYT	11	1	0	12	4	1	18	1.6	3	3.4	11	1.6	10.7	12.7	17.9	12.8	17.8	20.0	15.8	11.9	27.7	30.9	35.5	39.9	4.2	61	ND
Es2.181	d2	1-18	3-10/4-23/1-7	5	γ1	DADLSANSGWFDP	13	0	3	18	5	κ	3-15	1	QQYNDWPRT	9	1	1	10	4	1	19	0.3	0.6	0.5	12	0.3	12.2	12.8	20.3	14.6	20.2	105.1	18.2	20.6	9.9	10.7	13.1	NS	0.1	98	ND
Es2.182	d2	1-3	3-22	6	γ1	PDSSGFYGVHYYYAMDV	17	1	2	24	7	κ	2D-28/2-28	4	MQALQSLT	8	0	0	7	3	1	19	0.3	0.4	0.4	11	9.3	11.3	8.6	9.2	13.1	12.1	10.9	14.3	11.8	20.2	21.1	21.5	22.0	4.5	21	ND
Es2.188	d2	4-4	2-2/2-8	4	γ1	KVLGYTSGWTLGHFDY	16	2	1	28	8	κ	1-5	4	QQYHSYSLT	9	1	0	5	2	3	18	0.4	0.9	0.6	11	0.4	17.1	12.2	12.1	21.5	19.5	16.7	17.4	22.6	19.1	17.2	28.7	24.9	4.4	4	ND
Es2.190	d2	3-30/3-33	1-1	4	γ1	DQGGVQLWTFDY	12	0	2	24	7	κ	3-11	4	QQRASGPLT	9	1	0	11	4	1	18	0.3	0.8	0.6	11	3.7	10.0	8.1	10.0	5.7	7.9	4.9	9.6	12.7	16.8	13.9	16.3	12.3	4.8	25	ND
Es2.202	d2	3-33	3-22	3	γ1	TWWFYNSSGYAASGAFDI	18	0	1	23	7	κ	3-20	2	QHYGSSPPYT	10	1	0	13	5	1	19	0.4	0.5	0.4	11	4.9	12.2	9.2	14.4	12.1	10.3	8.0	17.1	14.9	7.5	7.7	8.5	6.0	3.8	26	ND
Es2.217	d2	4-4	3-22/3-16	6	γ1	ANERSYYDTSGYAQYYYVMDV	21	1	3	16	4	κ	1D-39/1-39	1	QQSYSAPRT	9	1	0	17	6	1	18	0.3	0.4	0.5	11	6.6	22.6	11.8	16.3	13.6	15.2	13.1	18.2	29.7	14.4	15.4	17.4	13.1	4.4	26	ND
Es2.219	d2	5-Es1.1	1-14/2-21/2-15	6	γ1	HMIYLVDIEPVKGVYGMDV	19	2	3	25	7	κ	3-20	2	QQYGSSPS	8	0	0	9	3	3	19	17	15	ND	ND	ND	7.3	8.9	14.4	8.7	11.8	8.5	7.3	7.1	10.3	32.9	17.1	17.1	0.8	79	0.29
Es2.224	d2	4-39	3-10	5/4	γ1	HVGGGSGSSVDK	12	2	1	21	6	κ	1-5	1	HQYNSYTPT	9	1	0	13	5	1	18	0.5	0.5	0.6	11	3.5	19.2	14.8	19.4	38.0	26.9	16.2	19.8	21.7	13.4	11.5	15.6	10.2	4.4	19	ND
Es2.236	d2	1-18	3-22	6	γ1	GLFFYDSSGIEVYYYYGMDV	20	0	3	21	6	κ	3-20	1	QQYGSSPPT	9	0	0	14	5	1	19	0.3	0.3	0.4	11	5	9.5	7.8	12.1	9.4	13.0	12.7	9.6	15.3	21.4	27.4	48.7	54.7	4.4	19	ND
Es2.239	d2	4-4	2-21/3-9	6	γ1	ASEFTVKIAVTGYDNYYFAMDV	22	1	3	17	5	κ	1D-39/1-39	1	QQSYSAPRT	9	1	0	21	8	4	18	0.3	0.3	0.5	11	8.2	9.6	7.8	10.9	7.7	9.3	10.3	8.9	15.6	12.6	18.8	23.5	33.0	4.8	6	ND
Es2.278	d2	3-30	5-12	4	γ1	DSRGGYDSILEY	12	1	3	25	8	κ	3-11	2	QQRANWPPS	9	1	0	16	6	2	19	0.4	0.3	0.5	14	4.3	5.8	9.0	6.7	6.4	15.2	6.8	6.3	7.6	10.0	13.5	22.6	32.3	4.9	0	ND
Es2.280	d2	3-48	3-22	3	γ1	NYYDSSGYYSGAASDI	16	0	2	15	4	κ	3-11	2	QQRSSWPPDT	10	1	1	11	4	2	15	11	12	ND	0.3	ND	4.9	4.5	5.2	3.8	6.6	5.0	7.2	4.5	9.0	10.1	NS	12.2	0.1	55	ND
Es2.288	d2	4-31	3-3	4	γ1	EGEWSRVFDY	10	1	3	21	6	κ	3-15	2	QQNYRWPQHT	10	2	0	11	4	1	18	0.3	0.4	1	12	6	10.5	13.9	16.4	9.1	64.6	14.9	9.0	24.2	10.1	11.6	17.9	31.4	4.8	15	ND
Es2.312	d2	1-18	6-19	5	γ1	DGALAGTRGWFDP	13	1	2	16	5	κ	3-20	5	QQYGSSPGIT	10	0	0	13	5	1	16	0.4	0.4	ND	15	0.5	260.9	92.9	16.5	21.8	23.9	NS	NS	NS	12.8	22.2	NS	NS	0.1	0	ND
Es2.316	d2	3-30	1-14/1-20/1-7	3	γ1	DIEGGYNSAFDI	12	0	3	11	3	κ	3-11	2	QQRGIWPPT	9	1	0	6	2	1	19	0.3	0.3	0.5	11	3.2	8.2	6.5	9.4	9.6	7.8	7.8	11.6	8.9	7.0	9.6	15.4	20.9	4.6	0	ND
Es2.317	d2	4-4	3-9	5	γ1	RAFDILTGPGITWFDP	16	1	2	19	6	κ	1D-39/1-39	2	QQSYSTPHT	9	1	0	15	6	2	19	0.4	0.4	0.4	11	0.3	10.0	8.2	14.4	25.7	9.5	8.8	26.9	11.3	11.1	30.0	26.1	38.3	4.8	9	ND
Es2.321	d2	3-74	2-21	4	γ1	DEYCGGDCYFDY	12	0	4	7	2	κ	1-27	1	QKYNSAPLT	9	1	0	3	1	1	16	17	16	ND	0.7	ND	6.3	5.8	13.9	5.5	9.4	7.4	8.1	8.0	7.3	21.2	10.6	10.2	0.1	43	50
Es2.325	d2	3-23	6-19	4	γ1	MGYNSGWAPIVPYHFDY	17	1	1	18	5	κ	3-11	4	QQRVNWPLT	9	1	0	11	4	2	15	11	11	ND	0.4	ND	4.2	4.2	7.8	4.5	8.2	4.7	3.8	7.5	11.7	16.4	19.2	22.6	0.1	99	0.037
Es2.326	d2	1-2	2-15/2-21	6	γ1	QRTFVVVVAATGFYYHAMDV	20	2	1	17	5	κ	3-11	5	QQSNNGGSIT	10	0	0	9	3	1	18	0.4	0.4	0.4	11	0.4	10.0	6.7	13.2	7.2	12.0	8.5	26.4	14.1	8.3	12.1	17.6	25.0	3.6	35	ND
Es2.329	d2	4-59	2-2/2-8	5	γ1	RNMRPTPRFDN	11	3	1	43	13	κ	1D-39/1-39	1	QQSHSTPWT	9	1	0	18	7	1	19	0.5	0.3	0.5	14	0.5	8.5	7.6	12.1	10.1	10.1	9.8	24.9	15.9	6.2	7.1	14.8	19.4	4.9	0	ND
Es2.336	d2	3-30	3-16	4	γ1	SLRAYYFDQ	9	1	1	14	4	κ	1-5	1	QQYNSDSLWT	10	0	1	9	3	1	18	0.5	0.4	0.4	14	0.4	7.0	5.1	11.7	6.7	11.2	5.3	15.2	9.3	4.8	7.1	9.7	15.7	4.6	0	ND
Es2.342	d2	1-18	6-13	5	γ1	DSWEVASWFDP	11	0	3	22	7	κ	3-20	1	QQYGSSPLT	9	0	0	14	5	4	19	0.4	0.4	0.5	14	0.4	15.0	9.0	25.7	14.3	19.3	25.2	24.8	21.0	9.7	12.0	18.4	NS	0.1	100	ND
Es3.102	d3	4-4	3-3	4	γ1	LEIFGVIVPPATDY	14	0	2	31	9	κ	1-17	1	LQHNSYPRT	9	2	0	11	4	1	13	2.1	2.5	ND	11	ND	47.5	66.5	69.6	113.2	51.9	53.5	53.6	118.0	648.4	994.0	NS	NS	0.1	ND	ND
Es3.108	d3	4-59	5-5/5-18	5	γ2	EVSAGHTYGYHWFDS	15	2	2	19	6	κ	2D-28/2-28	1	MQALQTPWT	9	0	0	7	2	1	14	14	14	ND	ND	ND	8.3	8.6	12.1	11.5	8.8	9.6	7.0	6.8	9.0	13.8	16.4	17.9	0.2	99	0.044
Es3.110	d3	3-30/3-30-3	1-26/2-8	4	γ1	LWDLMPDY	8	0	2	28	9	λ	2-8	3	YSYAGSNSLM	10	0	0	16	5	1	11	0.3	0.5	0.3	15	6.6	8.1	7.9	7.5	11.4	6.7	8.5	7.5	9.8	12.8	13.3	20.7	29.7	4.3	13	ND
Es3.111	d3	3-30	3-3	6	γ4	DSQLYYDFWSVYKGYMDV	18	1	3	24	7	κ	3-11	3	QQRTSWPLT	9	1	0	16	6	1	13	0.4	0.3	0.6	14	5.4	7.9	7.2	7.6	12.3	8.1	7.4	7.2	8.5	20.6	17.3	34.6	29.2	4.6	4	ND
Es3.112	d3	4-4	3-10	3	γ2	ALEFYSHRSLYVWAFDM	17	2	2	18	5	κ	1D-39/1-39	1	QQSYSAPRT	9	1	0	14	5	1	12	0.4	0.4	0.5	14	7.4	10.1	10.5	9.2	13.5	11.2	7.3	10.2	10.3	21.5	30.0	41.0	54.7	4.4	33	ND
Es3.125	d3	4-39	5-24	2	γ2	HGLARATIWNFDL	13	2	1	18	5	λ	1-51	3	GTWDSSLGAWV	11	0	1	15	5	1	11	0.4	0.4	0.5	14	4	10.8	11.3	22.6	10.4	16.8	9.7	9.4	14.7	24.5	30.3	29.0	61.4	4.4	14	ND
Es3.126	d3	3-30/3-30-3	3-16	4	γ2	DTMEDFAWGSDLSVTHEDY	19	1	6	8	2	κ	3-20	1	QHYGSTPWT	9	1	0	11	4	1	9.7	0.3	0.5	0.4	13	0.7	14.2	15.6	19.3	29.0	20.4	15.5	19.6	18.1	NS	NS	NS	69.0	0.1	2	ND
Es3.127	d3	3-21	3-16	6	γ1	DAYEFWTTYSSHHYFYYMDI	20	2	3	21	6	λ	1-40	3	QSFDTSLSRGV	11	1	1	11	4	1	12	0.3	0.3	0.5	15	3.9	10.3	8.6	14.0	8.1	11.1	8.5	14.0	12.1	10.7	10.2	14.7	20.3	4.5	11	ND
Es3.128	d3	3-23	1-26/5-12/4-23	5	γ1	CPTWVAIHSPLDS	13	1	1	20	6	κ	3-20	1	QQYHNLPQT	9	1	0	20	7	1	12	0.3	0.3	0.5	15	0.4	11.7	11.7	9.3	10.0	9.6	7.4	11.3	9.9	12.5	15.6	34.0	37.5	0.8	16	ND
Es3.130	d3	4-39	3-3	4	γ1	HWVAMGYDVGSGYYTRGFDQ	20	2	2	19	5	κ	3-20	1	QQNGHSPPWT	10	1	0	13	5	1	12	0.7	0.8	1.5	15	1.1	6.5	9.4	10.4	6.3	9.2	10.1	12.8	14.1	14.8	16.1	25.2	34.9	4.4	23	ND
Es3.133	d3	4-61	5-5/5-18/3-10	5	γ3	DPYGGWFAP	9	0	1	20	6	κ	3D-15	1	QQYTNWPPWT	10	0	0	8	3	1	11	0.3	0.5	0.4	13	3	16.7	15.9	22.9	13.7	13.8	13.7	21.8	19.3	22.3	41.9	66.1	83.2	4.5	16	ND
Es3.135	d3	3-21	1-1/7-27	3	γ1	ENNWGAAFDI	10	0	2	18	6	κ	1-5	2	QQYNSYPYT	9	0	0	9	3	1	12	0.3	0.3	0.9	14	4	10.9	12.6	15.8	10.1	7.8	14.7	8.8	13.3	33.7	30.2	62.6	70.2	ND	0	ND
Es3.137	d3	4-39	5-12	4	γ1	RVGLRHFDH	9	4	1	32	10	λ	1-51	3	GTWDSSLSVWV	11	0	1	11	4	1	11	0.3	0.3	0.4	13	3.6	5.8	5.5	3.7	5.3	10.9	4.8	5.2	5.4	22.3	24.6	32.4	57.1	4.2	5	ND
Es3.141	d3	3-23	6-19	4	γ2	DNLWQWLGFDY	11	0	2	27	8	λ	2-14	1	SSFSSSSTPYV	11	0	0	16	5	1	13	0.4	0.4	0.5	14	2.4	7.3	8.6	5.1	5.6	9.0	5.5	6.8	6.1	11.7	11.6	15.8	23.4	5.0	15	ND
Es3.142	d3	1-69	3-10	6	γ1	GLHWFGELNHYGMDV	15	2	2	26	8	λ	2-23	3/2	CSYAGSNNPVL	11	0	0	14	5	1	12	0.4	0.9	0.6	13	4.7	12.8	15.2	10.7	6.1	18.6	6.4	7.7	11.0	19.0	19.1	34.4	40.4	4.3	9	ND
Es3.143	d3	4-31	7-27	4	γ1	GMIWGRQNFDS	11	1	1	17	5	λ	2-11	3	CSFAGTYTV	9	0	0	13	4	1	14	2.1	1.6	ND	12	ND	12.4	10.5	18.7	17.8	24.6	20.1	15.1	11.6	46.2	35.5	50.6	64.6	4.4	10	ND
Es3.147	d3	4-31	1-26	4	γ4	GGEWERLFDY	10	1	3	25	8	κ	3-15	1	QQYNNWPPWA	10	0	0	17	6	1	13	0.4	0.3	0.4	12	4.1	11.3	10.9	8.1	6.7	13.5	7.9	8.7	10.7	14.4	15.4	22.9	35.9	4.7	18	ND
Es3.149	d3	4-39	3-9	4	γ2	RNLHYFDY	8	2	1	36	11	κ	1-9	5	QQLDSYSSIT	10	0	1	19	7	1	12	0.5	0.4	0.4	13	3.4	9.5	12.0	7.4	6.0	18.9	8.1	8.1	21.3	10.9	10.0	18.9	27.5	ND	21	ND
Es3.150	d3	1-69/1-69D	3-10	6	γ1	TLLWFGESNHFGMDV	15	1	2	25	7	κ	2D-28/2-28	1	MQGLQIPRT	9	1	0	14	5	1	14	0.4	0.8	1	14	5.9	1.1	0.9	0.1	0.4	0.4	0.8	0.7	60.8	10.8	12.6	20.0	26.5	3.8	24	ND
Es3.152	d3	4-61	2-15	6	γ1	AAVYCSGGSCYYYYGMDV	18	0	1	19	5	κ	3-15	4	QQYNNWPPLT	10	0	0	5	2	1	9.8	0.4	0.5	0.5	13	2.8	10.1	7.6	9.7	9.6	9.2	12.5	11.6	7.6	15.1	17.7	25.4	32.8	4.3	13	ND
Es3.155	d3	4-31	3-9	5/4	γ2	GSEWNSYFEF	10	0	2	23	7	κ	3D-15	3	QQSHTWPLFT	10	1	0	16	6	1	13	0.5	0.4	ND	13	3.7	5.1	5.2	6.5	6.8	7.8	6.5	15.7	6.3	14.2	15.7	26.1	32.6	4.4	2	ND
Es3.157	d3	3-11	7-27	4	γ1	NGDYFDY	7	0	2	9	3	κ	1-5	1	QQYNSYSN	8	0	0	4	2	3	12	12	16	ND	ND	ND	8.8	9.5	12.4	7.2	8.7	9.0	8.4	8.7	8.3	10.5	11.3	11.2	0.1	38	50
Es3.160	d3	4-39	4-23/4-17	1	γ1	HDYGFFFQY	9	1	1	30	9	κ	3-15	1	QQYYYWPRT	9	1	0	9	3	1	13	0.4	0.5	0.5	15	0.4	6.7	8.6	7.7	8.5	12.9	7.4	18.7	8.5	14.7	15.0	18.1	26.2	4.8	15	ND
Es3.163	d3	1-69	1-1/2-2/2-8	6	γ1	DPASLYTLSYNEMDV	15	0	3	17	5	λ	1-40	3	QSYDNSLIPSWV	12	0	1	17	6	1	12	0.5	0.5	0.6	14	5.9	7.9	7.3	14.1	12.4	14.3	11.8	14.2	12.6	20.1	24.2	35.1	50.5	4.6	19	ND
Es3.167	d3	4-30-4	3-22	4	γ1	STFYYETISEGVFMMPGIIDF	21	0	3	19	5	κ	3-20	1	QQYGSSPGT	9	0	0	10	4	2	14	0.6	1.3	ND	15	1.4	18.4	7.7	24.6	19.0	20.9	21.8	18.1	14.4	14.9	16.9	24.8	30.1	4.7	13	ND
Es3.170	d3	4-34	2-15	3	γ4	YCSAGHCGYKDAFEI	15	2	2	24	7	κ	1D-39/1-39	5	QQSYSAPSIT	10	0	0	19	6	1	15	14	16	ND	1.1	ND	21.9	21.3	28.2	23.4	31.5	27.2	39.0	183.7	13.6	118.7	22.3	22.5	0.5	99	9.78
Es3.174	d3	3-43	6-19	6	γ1	DIYLRRDYRSGWYAGSMDV	19	3	3	16	5	λ	1-47	3	ATWDDSLSGRGVL	13	1	2	17	4	1	12	0.4	0.4	0.4	14	0.4	30.5	6.1	19.9	18.1	9.3	35.5	9.8	12.0	21.0	22.4	28.0	42.6	4.5	16	ND
Es3.180	d3	1-18	3-3/3-22	1	γ4	DAEWFSNSGYGDS	13	0	3	29	9	κ	3-15	1	QQYNSWPWT	9	0	0	12	7	1	14	0.4	3.3	0.7	15	0.4	8.7	10.0	10.9	8.8	9.7	7.8	14.4	13.1	12.4	13.9	18.6	38.7	0.2	97	ND
Es3.182	d3	4-39	3-16	3	γ1	RVGHLGVFDI	10	2	1	27	8	κ	1-12	4	QHANSFLPLT	10	1	0	18	7	1	13	0.8	2.5	ND	13	ND	7.0	6.8	13.9	7.8	16.6	11.7	7.3	14.5	2.3	2.4	3.4	4.9	4.6	29	ND
Es3.185	d3	4-4	2-15	5	γ1	AHTYYSGGTYYWGIDP	16	1	1	21	6	κ	1D-39/1-39	1	QQSYNAPRT	9	1	0	21	7	1	13	0.4	0.5	0.5	13	8.2	22.3	6.9	11.7	6.6	19.9	11.2	25.0	7.9	13.3	15.0	19.4	26.1	4.7	31	ND
Es3.187	d3	4-31	3-3	5	γ2	SYSEWLLSFDP	11	0	2	33	10	κ	3-20	1	QQFGSSPWT	9	0	0	16	6	1	12	0.3	0.5	0.5	11	3.8	12.9	10.2	16.3	9.0	10.3	9.4	18.5	21.4	18.4	19.5	31.6	50.1	4.9	0	ND
Es3.189	d3	4-31	3-10	4	γ1	EGGSGYTYGSYFFDY	15	0	2	24	7	κ	3-11	5	QQRSNWPPVST	11	1	0	8	3	1	14	13	12	ND	ND	ND	7.0	6.8	11.4	6.9	12.3	9.9	6.1	9.5	16.9	21.3	28.1	28.5	0.6	36	50
Es3.190	d3	4-39	3-3	4	γ1	HDWRVVFDY	9	2	2	23	7	κ	3-20	1	QQYGLFWT	8	0	0	9	3	1	12	0.4	0.4	0.4	12	4.3	12.0	6.2	10.3	6.2	13.2	9.9	18.3	18.5	13.4	15.2	21.9	29.8	4.5	2	ND
Es3.192	d3	4-39	3-10	2	γ2	RSFWYFDL	8	1	1	23	7	λ	1-40	1	QSYDNLSGYV	10	0	1	17	6	1	12	0.5	0.4	0.5	12	3.3	16.7	5.6	7.7	6.6	15.1	14.2	27.6	8.8	14.8	17.2	23.1	25.5	4.5	2	ND
Es3.202	d3	4-39	2-8	4	γ1	HRYDYHSDTGGFDY	14	3	3	22	6	κ	1-5	1	QQYDSLWT	8	0	1	12	5	1	13	0.4	0.5	0.4	13	7.3	7.1	5.0	6.4	6.6	7.3	6.4	7.2	6.4	9.9	12.9	20.0	22.1	4.9	2	ND
Es3.223	d3	3-21	5-12	4	γ1	DSGWLRPDYFDY	12	1	3	15	5	κ	1-5	1	HHYNSYSPWT	10	2	0	10	4	1	13	0.4	0.4	0.4	12	4.3	5.7	4.9	7.2	5.5	6.3	5.7	6.8	6.3	15.2	17.3	22.2	35.1	4.6	0	ND
Es3.321	d3	4-39	3-22	5	γ1	HIVVTLNWFDP	11	1	1	12	4	λ	1-51	3/2	GTWDSSLSAVV	11	0	1	5	2	3	14	0.3	0.4	ND	10	ND	10.3	8.4	12.2	14.5	10.2	6.2	16.1	10.1	14.2	22.5	29.9	50.2	0.1	3	ND
Es4.205	d4	3-64D	5-12	4	γ1	VASQVWISNYFDY	13	0	1	18	5	κ	1-5	5	QQYNSYSVT	9	0	0	12	4	2	11	0.4	0.4	0.6	14	2.6	10.1	6.2	9.4	15.7	8.4	6.2	23.4	16.3	11.8	15.2	21.8	40.3	4.8	12	ND
Es4.207	d4	3-74	2-21	4	γ1	DAYCGGDCYSDY	12	0	3	13	4	κ	3-20	5	QQYGGSSIT	9	0	0	11	4	5	11	11	12	ND	ND	ND	5.4	5.3	8.2	4.7	5.7	5.7	7.1	6.4	10.5	14.3	14.7	13.9	0.1	87	0.36
Es4.208	d4	4-59	3-16	5	γ1	GETWGRWFDP	10	1	2	19	6	κ	3-11	2	QQRSNWLYT	9	1	0	10	4	1	10	0.3	0.4	0.4	12	2.1	8.5	6.4	9.6	7.6	11.9	7.2	12.8	10.2	15.5	16.8	31.9	35.8	4.8	0	ND
Es4.210	d4	4-59	3-3	5	γ1	QRDYDFWSAESIAWARWFDP	20	2	4	26	7	κ	4-1	2	QQYYSTPYT	9	0	0	10	4	1	9	0.9	0.4	0.5	13	3.4	12.4	10.7	12.8	16.5	16.2	10.4	16.7	16.1	19.1	21.2	34.8	48.1	4.3	ND	ND
Es4.211	d4	4-31	3-3	3	γ1	RGEWLVGFDI	10	1	2	21	6	κ	3-20	1	QQYAGLVWT	9	0	0	10	4	1	9.8	0.5	0.7	0.6	13	3.8	10.2	5.5	11.4	5.2	13.8	6.7	17.8	8.2	14.2	16.4	23.0	36.4	4.3	11	ND
Es4.216	d4	4-59	3-3	5	γ1	GDWSGYLHWFDP	12	1	2	9	3	κ	1-NL1	1	QQYYSPPPT	9	0	0	8	3	3	9.5	0.3	0.4	0.4	14	3.9	8.9	4.9	10.0	4.8	11.4	5.5	14.9	6.0	15.8	16.4	22.9	44.5	4.5	0	ND
Es4.218	d4	3-66	3-10	6	γ1	DRRGFYGPGSYKDGMDI	17	3	3	25	7	κ	1-5	2	QQYNSYPYT	9	0	0	14	5	4	15	0.5	0.5	ND	14	1.7	8.3	6.9	11.2	16.1	11.2	11.2	16.1	10.9	15.0	19.1	27.2	44.0	3.7	0	ND
Es4.229	d4	4-30-4/4-31	6-25	4	γ1	GDNSGFVHYFDY	12	1	2	19	6	κ	1-NL1	4	QQYYTTVT	8	0	0	18	7	1	11	0.4	0.4	0.4	14	3.4	6.9	5.2	15.7	13.0	7.5	12.9	10.2	7.4	12.0	14.4	17.3	34.4	4.9	44	ND
Es4.233	d4	3-33	2-8	5/4	γ1	WMVPAPGSLGL	11	0	0	16	5	κ	3-15	1	QQYSNWPPWT	10	0	0	16	6	3	11	0.5	0.5	0.5	14	3.3	6.5	6.0	8.5	7.2	10.3	7.8	12.2	9.4	10.4	12.5	17.1	25.2	3.8	0	ND
Es4.239	d4	4-59	3-3	6	γ1	NRLGGYYDVWSGDYYSGLDV	20	1	3	18	5	κ	2D-28/2-28	1	MQALQTPWT	9	0	0	3	1	1	12	0.4	0.4	0.7	13	1	25.6	27.1	29.5	28.7	40.9	25.3	55.2	37.6	29.0	44.9	64.9	100.2	0.1	0	ND
Es4.247	d4	4-30-4	6-19	6	γ1	DLMFAGSDPGYGSGWSKNYYYLMDV	25	1	3	12	3	κ	1D-39/1-39	2	QQSYSTPLYT	10	0	0	9	3	1	11	0.4	0.4	0.5	13	0.4	15.1	11.9	13.8	9.6	21.4	10.5	27.6	8.0	13.1	17.8	27.3	33.9	0.1	9	ND
Es4.248	d4	3-33	3-22	6	γ1	VDSSGFYGANYYYAMDV	17	0	2	18	5	κ	2D-28/2-28	1	MQALQTPPWT	10	0	0	6	2	2	11	0.4	0.4	0.5	14	8	11.0	10.8	17.5	9.3	13.8	11.7	17.4	11.2	16.1	20.9	24.6	36.7	4.7	0	ND
Es4.252	d4	3-30	6-19/6-13/6-25	5/4	γ1	DHGRGYRAYMDH	12	4	2	17	5	κ	3-11	4	QQRANWPLT	9	1	0	14	5	2	12	0.4	0.4	0.6	13	3.5	18.4	7.3	25.6	8.3	19.9	11.0	25.1	10.5	16.0	21.9	31.8	45.1	4.6	3	ND
Es4.261	d4	3-33	2-15/2-21	4	γ1	DIAGSCGGGRCGGFDN	16	1	2	20	6	κ	3-15	2	QQYSNWYT	8	0	0	7	3	5	16	0.5	0.4	ND	14	5.6	10.0	7.1	9.2	5.7	9.3	8.1	15.5	9.6	11.0	13.6	16.6	27.0	4.9	25	ND
Es4.263	d4	4-31	6-19	4	γ1	GEQWLGHFDY	10	1	2	19	6	κ	3-20	2	QQYGSSPIYT	10	0	0	8	3	4	11	0.4	0.4	0.7	13	3.9	6.5	8.3	9.0	9.7	8.1	7.7	7.3	9.8	16.2	17.7	28.1	38.4	4.6	3	ND
Es4.268	d4	3-74	2-21	4	γ1	DAIRCGGDCYPDY	13	1	3	21	6	κ	4-1	4	HQYYTTPLT	9	1	0	8	3	1	10	8.9	17	ND	ND	ND	16.5	20.1	21.3	21.6	21.3	19.8	25.5	20.1	13.0	19.1	17.8	22.9	0.2	38	50
Es4.269	d4	1-69	3-22	5/4	γ1	GQTYYYEN	8	0	1	21	7	κ	3-11	2	HQRSNWPLYT	10	2	0	10	4	1	13	0.4	0.5	0.9	15	0.5	3.7	3.0	5.0	3.7	4.6	3.5	4.4	5.0	7.7	9.5	11.2	19.9	4.9	5	ND
Es4.271	d4	4-59	2-2/2-15	3	γ1	GMTWAAAFDI	10	0	1	20	6	κ	3-11	1	QQRTNWLWT	9	1	0	8	3	1	11	0.6	0.6	0.8	13	4.5	5.9	6.8	7.3	7.2	7.2	7.0	6.3	8.8	13.1	11.1	17.8	21.8	4.7	ND	ND
Es4.273	d4	3-64D	2-8	4	γ1	PFVLYAVSYYFDH	13	1	1	14	4	κ	1-NL1	2	QQYYSSPAYT	10	0	0	14	5	1	10	0.5	1.3	0.7	12	1.5	7.4	7.4	11.4	9.2	12.6	6.7	11.4	10.9	8.0	9.5	9.9	14.5	4.5	4	ND
Es4.280	d4	4-31	3-3	4	γ1	REIWSGYYVY	10	1	1	18	6	κ	1-NL1	2	QQYYSIPYT	9	0	0	11	4	1	11	0.4	0.4	0.4	14	4	18.0	4.1	26.8	3.9	21.2	3.8	17.6	5.0	8.8	10.2	9.5	12.3	4.7	0	ND
Es4.282	d4	4-31	3-22	5	γ1	GDSRGYVHWFDT	12	2	2	24	7	κ	1-NL1	4	QQYYSTPLT	9	0	0	13	5	5	11	0.3	0.3	0.4	14	3.5	6.2	5.0	5.2	6.1	6.7	5.4	6.5	7.9	9.6	11.1	11.2	12.4	4.7	16	ND
Es4.283	d4	3-30	1-7	4	γ1	DRRQGYTLELDY	12	2	3	22	7	κ	3-11	5	QQRGKWPPA	9	2	0	15	6	2	11	0.4	0.4	0.4	12	4.2	13.3	7.3	14.1	8.1	12.7	5.5	10.2	9.4	33.8	59.6	44.3	40.4	4.1	0	ND
Es4.287	d4	3-7	1-1	4	γ1	GLTTGAFRWAY	11	1	0	20	6	κ	1D-39/1-39	4	QQSYNTPHT	9	1	0	15	5	3	15	0.6	0.4	ND	13	0.5	36.3	9.1	36.7	15.9	33.4	7.7	28.9	17.8	8.4	10.6	10.8	9.7	0.1	13	ND
Es4.403	d4	3-7	1-1/3-3/1-20	4	γ1	ALTTGPFRFDY	11	1	1	25	8	κ	1D-39/1-39	2	QQSYNSPQT	9	0	0	20	7	3	15	0.5	0.4	ND	13	0.5	118.2	13.3	45.2	12.9	77.5	7.0	45.4	20.7	10.9	15.9	13.6	12.7	0.1	11	ND
Es4.417	d4	4-59	3-22	5/4	γ1	ESGGYYEF	8	0	2	16	5	κ	3-15	1	QHYYNWPPWT	10	1	0	9	3	2	12	0.5	0.5	0.5	12	4.7	32.7	15.7	32.4	15.6	33.3	24.4	29.9	33.1	12.7	11.4	12.2	11.9	4.4	3	ND
Es4.431	d4	3-23	2-8	4	γ1	VVHRCTTNTCFRGADN	16	3	1	23	7	κ	1D-39/1-39	2	QQSYTTLMYT	10	0	0	18	7	3	13	13	11	ND	ND	ND	5.9	5.5	6.6	4.6	7.0	4.7	4.6	6.1	9.9	11.0	13.8	13.7	0.1	97	0.0007
Es4.452	d4	3-30	2-2	6	γ1	GGLVPSPIIGLDV	13	0	1	16	5	κ	1-9	4	QQLNSFPLS	9	0	0	16	6	9	13	13	12	ND	ND	ND	6.8	6.3	6.7	5.6	8.8	7.3	8.7	7.9	10.4	13.0	12.9	17.5	0.2	100	0.0025
Es4.492	d4	4-31	6-19	4	γ1	RQWVAFDY	8	1	1	28	9	κ	3-15	1	QQHHNWPPRWT	11	3	0	12	4	2	11	0.6	0.5	0.7	12	0.6	7.6	13.7	8.8	15.7	9.9	16.8	9.4	28.9	9.6	8.6	11.1	12.0	5.0	9	ND
Es4.493	d4	4-59	2-2	5	γ1	SGTSWHNFFDP	11	1	1	20	6	κ	3-15	1	QQYNNWLWT	9	0	0	6	2	2	10	0.5	0.4	0.5	10	1.5	13.8	3.7	12.1	6.3	11.7	3.9	9.2	9.4	13.3	7.1	21.3	10.1	4.6	20	ND
Es4.495	d4	4-31	4-11/4-4	4	γ1	EGDYSGQRGYFDY	13	1	3	22	7	κ	1D-39/1-39	2	QQSYRVPLYT	10	1	0	17	6	4	11	0.3	0.3	0.4	12	2.1	32.8	24.3	27.6	19.7	31.3	16.4	29.0	18.8	10.8	12.3	14.7	13.5	4.7	13	ND
Es4.503	d4	3-23	2-15/1-26/2-21	5	γ1	GWGWFDP	7	0	1	15	5	κ	3-15	1	QQYNHWPRT	9	2	0	8	3	1	8.8	0.3	0.4	0.4	12	1.2	59.2	38.2	33.9	63.9	62.0	43.9	35.4	62.4	24.0	41.2	59.5	32.7	0.8	3	ND
Es4.508	d4	5-51	2-2	6	γ1	HKVVVERGSIGFDYYYYAMDV	21	3	3	18	5	κ	4-1	4	QQYYSPPLT	9	0	0	11	4	3	11	0.6	0.7	1.9	13	0.5	25.9	20.4	31.0	16.5	27.3	28.3	39.0	17.7	12.9	15.8	16.4	20.0	0.2	43	ND
Es4.519	d4	4-59	3-10/3-16	2	γ1	TKRGYWYFDL	10	2	1	26	8	κ	3-11	2	QQRSHYYT	8	2	0	14	5	15	13	0.5	0.4	0.5	14	0.5	21.6	17.5	22.2	10.3	16.3	12.3	18.0	10.3	12.1	12.0	14.4	13.6	4.9	48	ND
Es4.520	d4	3-33	3-10	3	γ1	DQSPVPREQMVPNAFDF	17	1	3	19	6	κ	1D-39/1-39	1	QQSYTTSRT	9	1	0	15	6	2	10	0.5	0.5	0.4	12	0.5	29.9	25.9	31.0	19.6	33.4	15.8	46.0	18.5	13.2	13.6	17.0	16.1	0.1	16	ND
Es5.104	d5	4-59	4-23/2-21	5	γ1	IGDGGGWFDP	10	0	2	23	7	λ	2-23	3/2	CSYAGSYSNIGTYVL	15	0	0	10	3	5	11	11	16	ND	ND	ND	5.0	4.1	5.7	5.4	7.7	5.3	5.1	5.9	9.8	11.9	12.9	13.6	0.2	100	0.0137
Es5.114	d5	3-33	3-3	6	γ1	GLSGYRSGMDPKSYYYYAMDV	21	2	2	15	4	κ	1D-13	4	QQFNNYPLT	9	0	0	6	2	1	9.8	0.3	0.4	0.4	13	2.5	8.0	11.1	15.3	6.9	9.4	10.2	11.8	10.7	15.5	15.5	15.3	24.4	4.1	18	ND
Es5.116	d5	1-2	3-22	3	γ1	ADLQGSYSDSRGYAFDV	17	1	3	14	4	κ	2D-28/2-28	2	MQALQAPYT	9	0	0	10	3	1	10	0.3	0.3	0.4	14	6.5	6.1	7.2	9.1	6.0	6.4	5.2	8.2	8.1	6.3	8.1	6.5	8.5	5.0	29	ND
Es5.127	d5	5-51	2-2	5	γ1	VRGSLKVPAAVPFDP	15	2	1	12	3	λ	1-51	3/2	GTWDSSLSDVV	11	0	2	5	2	2	16	16	16	ND	0.6	ND	7.0	6.1	6.1	7.1	10.0	6.6	5.7	7.5	11.3	14.4	14.6	17.0	5.5	98	0.0006
Es5.133	d5	4-59	3-10	6	γ1	GGDYNYYYNGMDV	13	0	2	11	3	λ	1-40	3/2	QSYDSSLSAL	10	0	1	5	2	1	10	0.3	0.6	0.4	15	2.5	9.1	9.4	11.9	9.1	7.7	8.8	9.9	10.3	19.4	22.5	24.5	26.2	4.3	22	ND
Es5.210	d5	3-33	3-3	4	γ1	DFDTYDGSAYYPY	13	0	3	6	2	κ	3-15	2	QQYYYWYT	8	0	0	9	3	1	11	0.3	0.3	0.5	14	7	9.2	7.6	8.6	9.4	6.4	7.4	9.6	14.9	9.5	10.3	11.4	10.7	4.7	13	ND
Es5.223	d5	3-11	3-10	6	γ1	HLSMVRGVNSRNYAMDI	17	3	1	14	4	κ	1D-39/1-39	3	QQSYHTPFT	9	1	0	25	9	1	11	10	15	ND	ND	ND	7.8	6.3	7.9	6.1	9.2	5.3	5.2	4.7	9.5	11.6	9.9	12.4	0.2	100	0.0405

(-) and (+) indicate the numbers of negatively and positively charged amminoacids in the IgH and IgL complementary determining region (CDR3), respectively. MUT, number of somatic mutations; NS, no signal; ND, not determined.

Table S3. Cryo-EM data collection, refinement and validation statistics for the complex of HEV-CA P domain with Es1.327 Fab

**Data collection and processing**	
Magnification	105,000x
Voltage (kV)	300
Microscope	Titan Krios
Electron exposure (e-/Å2)	50
Defocus range (μm)	-1.0 to -3.0
Pixel size (Å)	0.86
Initial particle images (no.)	171045
Final particle images (no.)	106903
Map resolution (Å)	3,5
FSC threshold	0.143
**Refinement**	
Initial model used (PDB code)	2ZTN
Model composition	
Non-hydrogen atoms (no.)	5538
Protein residues (no.)	727
Ligands (no.)	2
B factors	
Protein (Å2)	80.86
Ligands (Å2)	78.08
R.m.s. deviations	
Bond lengths (Å)	0.007
Bond angles (°)	1.487
Validation	
MolProbity score	2.38
Clashscore	19.33
Poor rotamers (%)	3.21
Ramachandran plot	
Favored (%)	96.64
Allowed (%)	3.36
Disallowed (%)	0.0

Table S4. Cryo-EM data collection, refinement and validation statistics for the complex of HEV-CA P domain with Es4.431 Fab

**Data collection and processing**	
Magnification	105,000x
Voltage (kV)	300
Microscope	Titan Krios
Electron exposure (e-/Å2)	50
Defocus range (μm)	-1.0 to -3.0
Pixel size (Å)	0.86
Initial particle images (no.)	171045
Final particle images (no.)	106903
Map resolution (Å)	3,5
FSC threshold	0.143
**Refinement**	
Initial model used (PDB code)	2ZTN
Model composition	
Non-hydrogen atoms (no.)	5531
Protein residues (no.)	735
Ligands (no.)	2
B factors	
Protein (Å2)	68.01
Ligands (Å2)	64.21
R.m.s. deviations	
Bond lengths (Å)	0.008
Bond angles (°)	1.621
Validation	
MolProbity score	2.01
Clashscore	8.88
Poor rotamers (%)	3.70
Ramachandran plot	
Favored (%)	97.51
Allowed (%)	2.35
Disallowed (%)	0.14

Table S5. Cryo-EM data collection, refinement and validation statistics for the complex of HEV-CA P domain with Es5.127 and Es1.114 Fabs

**Data collection and processing**	
Magnification	105,000x
Voltage (kV)	300
Microscope	Titan Krios
Electron exposure (e-/Å2)	50
Defocus range (μm)	-1.0 to -3.0
Pixel size (Å)	0.86
Initial particle images (no.)	611380
Final particle images (no.)	207869
Map resolution (Å)	3,8
FSC threshold	0.143
**Refinement**	
Initial model used (PDB code)	2ZTN
Model composition	
Non-hydrogen atoms (no.)	8766
Protein residues (no.)	1172
Ligands (no.)	0
B factors	
Protein (Å2)	31.18
Ligands (Å2)	-
R.m.s. deviations	
Bond lengths (Å)	0.005
Bond angles (°)	1.046
Validation	
MolProbity score	2.25
Clashscore	12.61
Poor rotamers (%)	2.04
Ramachandran plot	
Favored (%)	94.06
Allowed (%)	5.24
Disallowed (%)	0.7

Table S6. Buried surface area calculated for the complex between HEV-CA P domain and Fabs.

**Es1.327 V_H**	**HEV-CA P domain**
594.2 sq Å (8.5%)	586.2 sq Å (7.8%)
T30 O	Q482 NE2
Y103 N	T483 O
T101 O	S488 N
H31 O	S488 OG
H31 ND1	S488 O
**Es4.431 V_H**	**HEV-CA P domain**
516.5 sq Å (7.4%)	476.3 sq Å (6.5%)
T31 O	Q482 NE2
H100 ND1	T483 O
H100 ND1	G486 O
H100 O	S488 N

The pair of residues forming hydrogen bonds between chains are indicated for each complex.

Table S7. Affinities of potent HEV neutralizing antibodies to HEV-CA and HEV-C1 proteins

	HEV-CA |g1a|			HEV-CA |g3-2712|			HEV-C1		
	ka ($M^{-1}s^{-1}$)	kd (s^{-1})	K_D (M)	ka ($M^{-1}s^{-1}$)	kd (s^{-1})	K_D (M)	ka ($M^{-1}s^{-1}$)	kd (s^{-1})	K_D (M)
Es1.327	1.0E+06	1.9E-04	1.9E-10	3.0E+05	1.7E-04	5.6E-10	n.d.	n.d.	n.d.
Es5.127	8.9E+05	2.4E-04	2.7E-10	6.8E+05	2.8E-04	4.1E-10	3.1E+05	4.9E-04	1.6E-09
Es4.431	8.4E+05	3.3E-04	4.0E-10	3.8E+05	3.1E-04	8.1E-10	n.d.	n.d.	n.d.
Es4.452	1.2E+05	4.5E-04	3.7E-09	2.7E+03	5.1E-04	2.0E-07	n.d.	n.d.	n.d.
Es1.117	1.8E+06	2.8E-04	1.6E-10	8.4E+05	2.7E-04	3.2E-10	n.d.	n.d.	n.d.

n.d.: not determined as no significant binding detected by surface plasmon resonance.

Table S8. Cryo-EM data collection, refinement and validation statistics for the complex of HEV-C1-CA P domain with Es5.127 Fab

**Data collection and processing**	
Magnification	105,000x
Voltage (kV)	300
Microscope	Titan Krios
Electron exposure (e-/Å2)	50
Defocus range (μm)	-1.0 to -3.0
Pixel size (Å)	0.86
Initial particle images (no.)	97277
Final particle images (no.)	56650
Map resolution (Å)	4.5
FSC threshold	0.143
